# Efficient Electrochemical Reduction of CO_2_ to Formate in Methanol Solutions by Mn‐Functionalized Electrodes in the Presence of Amines**

**DOI:** 10.1002/chem.202104377

**Published:** 2022-05-25

**Authors:** Francesca Marocco Stuardi, Arianna Tiozzo, Laura Rotundo, Julien Leclaire, Roberto Gobetto, Carlo Nervi

**Affiliations:** ^1^ University of Lyon, CNRS, CPE Lyon, INSA, ICBMS UMR 5246 69611 Lyon France; ^2^ University of Turin Department of Chemistry Via P. Giuria 7 10125 Turin Italy; ^3^ CIRCC via Celso Ulpiani 27 70126 Bari Italy; ^4^ current address: Chemistry Division Brookhaven National Laboratory Upton NY 11973–5000 USA

**Keywords:** carbon capture, carbon dioxide, electrocatalysts, electrochemistry, manganese

## Abstract

Carbon cloth electrode modified by covalently attaching a manganese organometallic catalyst is used as cathode for the electrochemical reduction of CO_2_ in methanol solutions. Six different industrial amines are employed as co‐catalyst in millimolar concentrations to deliver a series of new reactive system. While such absorbents were so far believed to provide a CO_2_ reservoir and act as sacrificial proton source, we herein demonstrate that this role can be played by methanol, and that the adduct formed between CO_2_ and the amine can act as an effector or inhibitor toward the catalyst, thereby enhancing or reducing the production of formate. Pentamethyldiethylentriamine (**PMDETA)**, identified as the best effector in our series, converts CO_2_ in wet methanolic solution into bisammonium bicarbonate. Computational studies revealed that this adduct is responsible for a barrierless transformation of CO_2_ to formate by the reduced form of the Mn catalyst covalently bonded to the electrode surface. As a consequence, selectivity can be switched on demand from CO to formate anion, and in the case of (**PMDETA**) an impressive TON_HCOO−_ of 2.8×10^4^ can be reached. This new valuable knowledge on an integrated capture and utilization system paves the way toward more efficient transformation of CO_2_ into liquid fuel.

## Graphical abstract

**Carbon cloth electrodes** were modified by attaching a manganese organometallic catalyst to the surface by covalent bonds. The system has been used for the electrochemical reduction of CO_2_ in presence of amines in methanol solution. The enhanced CO_2_ solubility and the use of pentamethyldiethylentriamine (PMDETA) induce change in chemical selectivity from CO to formate, with very high performances and high selectivity.

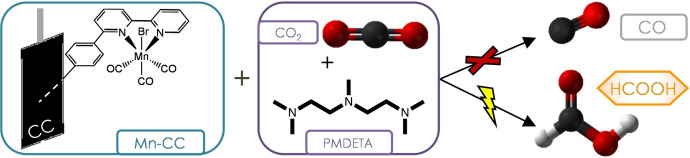

## Introduction

The development of efficient catalysts for the electrochemical reduction of CO_2_, a very active research topic, should not be envisaged in the sole framework of CO_2_ utilization, but rather as a brick of an entire carbon capture, utilization and storage (CCUS) value chain. Recent reports pointed out that an integrated approach, wherein the capture, utilization and storage technologies are designed to operate synergistically, may represent one of most effective options for viable and scalable GHG (Green House Gases) mitigation.[^1^, ^2^]

**Scheme 1:**
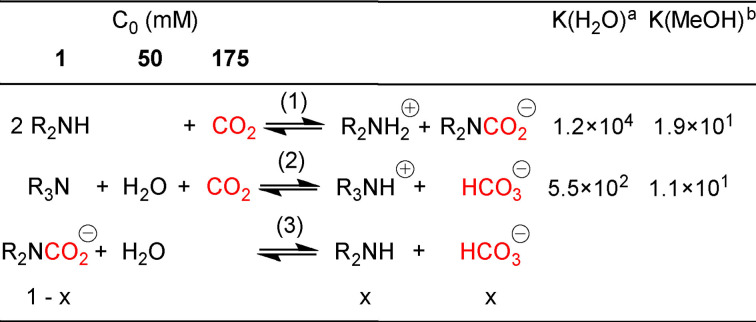
CO_2_ capture equilibria at work in our system, maximal concentration of species (C_0_, mM) and related binding constants. (1) carbamation; (2) carbonation; (3) carbamate hydrolysis. ^a^ from ref;^[8]^ ^b^ : from ref,^[3d]^ see Supporting Information S2

Post‐combustion CO_2_ capture is the most mature technology for flue gas treatment, and it is already implemented into existing power plants. To process diluted and low‐pressure streams, such as those emitted by fossil‐fired power plants, chemical absorption with aqueous amine solutions, called amine‐scrubbing, is the most appropriate technology.^[3]^ Amines spontaneously react with CO_2_ affording equilibrated mixtures of ammonium carbamates (Scheme 1, Equation (1)) and bicarbonate (in the presence of water, Scheme 1, Equation (2)). Captured CO_2_ can be released by thermally reversing these reactions, but the associated energetic cost is one of the major drawbacks.^[4]^

*In situ* direct transformation of captured CO_2_ (in the form of ammonium carbamate or bicarbonate) is a challenging alternative, which fits into the aforementioned process integration paradigm. Although most CO_2_ utilization processes reported to date operate from purified CO_2_, there has been a growing interest for the direct conversion of capture products in the past decade.^[5]^ These examples include the utilization of carbamates as metal extractants,^[3d]^ as vehicles for mineral carbonation,^[6]^ or their conversion into renewable fuels such as methanol.[^5^, ^7^]

The latter is a true transformation, not a utilization but it raises the issue of the electron source needed to produce hydrogen, itself required for CO_2_ reduction. Using renewable electrical energy (i. e. photochemical or electrochemical reductions^[9]^) for CO_2_ reduction surely represents a step further toward true sustainable CCUS but also an additional challenge. Yet, photochemical or electrochemical reduction strategies enabling to produce C1 and C2 chemicals from flue gases will certainly be one of the essential bricks of the next industrial revolution.^[10]^ The main products of CO_2_ electrolysis are usually CO, CH_4_, C_2_H_4_, formate, CH_3_OH and CH_3_CH_2_OH,^[11]^ which are valuable feedstocks for the chemical industry and for energy storage. A large array of transition metal complexes^[12]^ containing macrocyclic, (e. g. porphyrins, phthalocyanines, corroles and cyclams),^[13]^ polydentate, (e. g. 2,2’‐bipyridine (bpy), 1,10‐phenantroline (phen), etc.)^[14]^ and phosphine ligands (e. g. 1,2‐bis(diphenylphosphino)ethane (dppe), triphenylphosphine (PPh_3_), etc.)^[15]^ have been tested as molecular electrocatalysts in solutions, wherein CO_2_ was injected as a pure gas. Re‐ and Mn‐polypyridine complexes were shown to be among the most promising catalysts, displaying high reaction rates and selectivity. As a consequence, their reduction mechanisms were extensively explored. By fine‐tuning electronic properties and steric hindrance around the metal center, the selectivity and the activity of the electrocatalyst can be controlled.^[16]^ The availability of local proton sources is known to greatly impact these two parameters, potentially enabling to shift the CO_2_ reduction process from the production of CO to formate.[^16b^, ^17^] In particular, polypyridyl Mn(I) catalysts (e. g. [Mn(pdbpy)(CO)_3_Br] (pdbpy=4‐phenyl‐6‐(phenyl‐2,6‐diol)‐2,2’‐bipyridine) containing two acidic OH groups in proximity of the purported metal binding site for CO_2_ redox catalysis show enhanced catalytic activity towards HCOOH production.^[18]^

**Figure 1:**
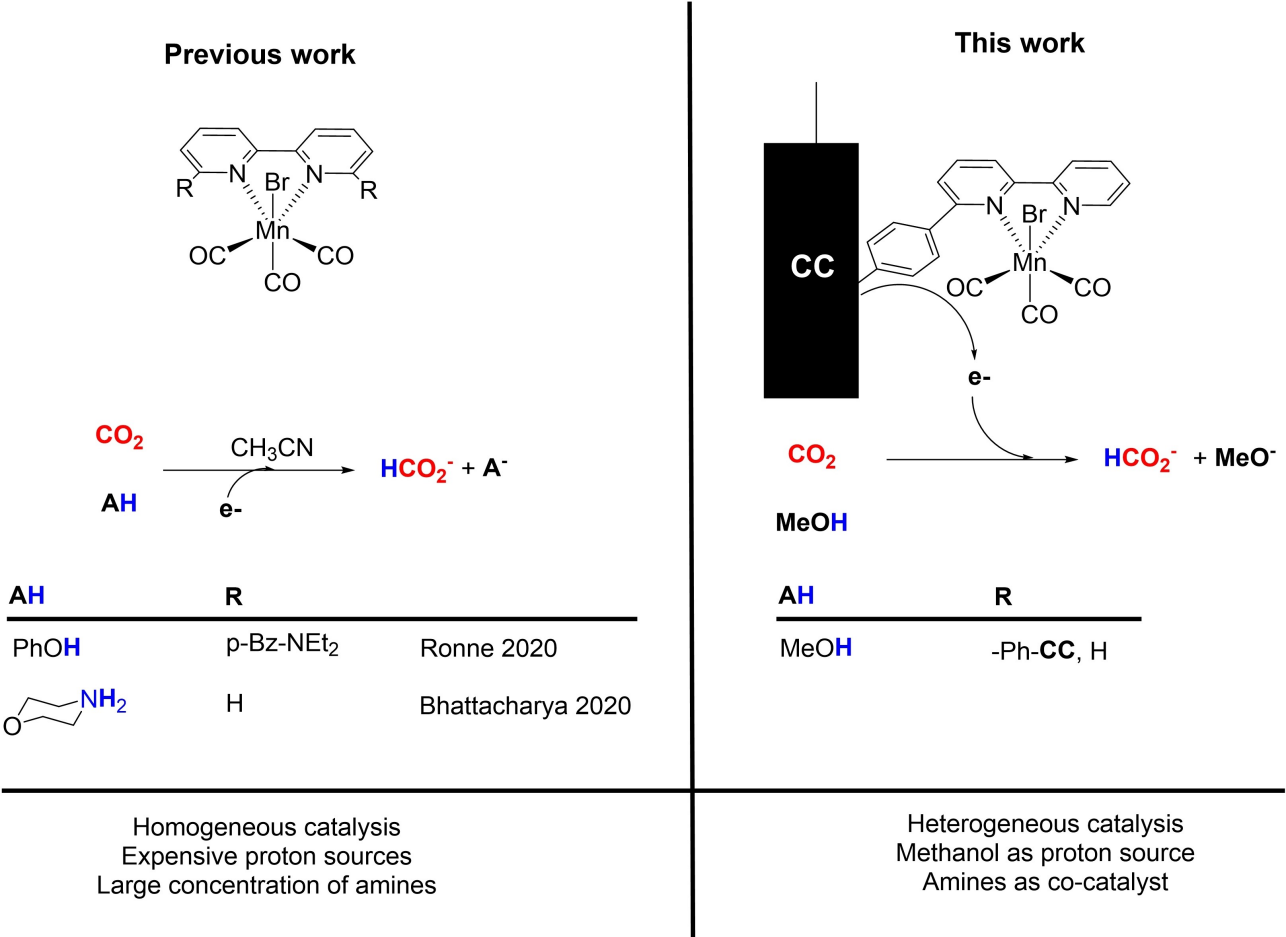
Schematic representation of the differences between previous integrated CO_2_ reduction systems in the presence of amines with homogeneous Mn bipyridyl complexes and the current work.

As mentioned earlier, examples of electrochemical CO_2_ reduction integrated to its absorption remain scarce.^[19]^ To our knowledge, only two studies to date reported the reduction of CO_2_ with a Mn‐based soluble electrocatalyst, in the presence of amines (in solution or bound to the metal chelating unit).^[19a]^ Amine moieties were proposed not only to provide binding sites, hence a reservoir of CO_2_ under the form of carbamates, but also to stabilize and promote the formation of the hydride catalytic intermediate (**HMn**), thereby favoring formate production instead of CO.[^19c^, ^20^] Contemporarily to our current work a third paper from Daasbjerg group^[21]^ appeared, regarding the use of amines with Mn catalysts, but in homogeneous solutions, different solvent, and different amine concentrations, which represent a nice complement to our heterogeneous approach. These amines, which are supposed to work as proton shuttle, were either introduced in large excess with respect the catalyst^[19a]^ or upon an elaborated synthetic procedure as a side‐arm of the Mn catalyst (Figure 1).^[20]^ Hydride transfer to CO_2_ requires a proton source, a role which was endorsed by acidic alcohols such as phenols or perfluoroalcohols. None of this class of sacrificial proton donors seems eligible for potential implementation into a cost‐effective industrial process, while water suffers from low CO_2_ solubility.

To enable the industrial utilization of CO_2_ for energy production, liquid fuels (i. e. formate) rather than gas precursors (i. e. CO) should be preferred for safety, storage and transportation reasons. In the same perspective of deployment, the immobilization of organometallic complexes onto a conductive support (via van der Waals interactions^[22]^ or by the formation of a covalent bond between the electrode surface and the intact transition metal catalysts^[23]^) is highly desirable. It enables to envisage a broader scope of solvents, including carbon capture media, and provides the reduction systems with increased durability, efficiency, recyclability and processability.^[23]^

Herein we investigate the role and impact of amines in millimolar concentration on the electroreduction of CO_2_ by a Mn bipyridyl complex (*fac*‐Mn(apbpy)(CO)_3_Br [apbpy=4‐(4‐aminophenyl)‐2,2’‐bipyridine)]) covalently bound to a Carbon Cloth (**CC**) surface. **CC** is a relatively cheap material of low electrical resistance and large surface area, widely exploited for the preparation of electrodes for low temperature fuel cells.^[24]^ During previous studies we benchmarked different strategies to attach intact organometallic species on carbonaceous electrode surfaces, such as a) oxidation of a terminal amine and alkylation with the carbon support,^[25]^ b) electrochemical reduction of diazonium salts and in situ generation of diazonium salts with formation of C−C bonds,^[25a]^ and c) functionalizing the catalyst with a thiophene moiety which is subsequently electropolymerized on the electrode surface.^[26]^ Alternative approaches involve adsorbing the catalysts onto electrode surface. The resulting material shows promising performances,^[22b]^ but there is some substantial risk of mechanical removal. Based on our experience, the diazonium salt methodology delivers heterogenized catalysts, which are by far more stable and displaying higher TOFs and TONs, more generally, better performances.^[27]^ Therefore, we adopted the diazonium salt methodology to anchor the catalyst on the carbon cloth.

**Figure 2:**
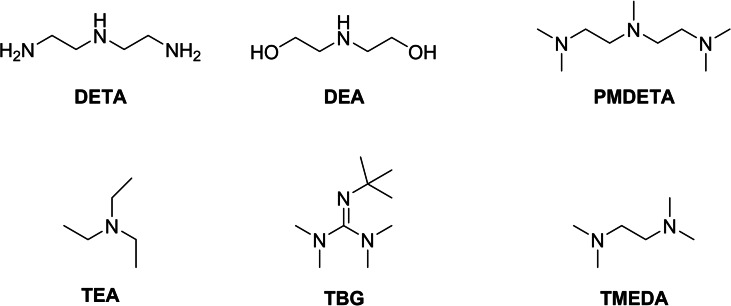
Panel of industrial amines (and guanidine) tested in this work.

The functionalized electrode (**Mn/CC**) was tested as a catalyst for CO_2_ electrochemical reduction in a three‐electrode cell with two gastight compartments, with methanol as a solvent. Its performance was studied in the presence of a panel of industrial amines (Figure 2) by means of Controlled Potential Electrolysis (CPE).

By a combination of theoretical and experimental investigations, we herein show that methanol acts at the same time as a carbon solvent, enhancing CO_2_ solubility compared to water, and as an affordable sacrificial proton source. In our system, amines rather play the role of homogeneous co‐catalysts or co‐factors during the reduction process. Combining a heterogeneous catalysis approach and a more accessible sacrificial proton source may also pave the way toward scalable capture and integrated electroreduction processes.

## Results and Discussion

*fac*‐Mn(apbpy)(CO)_3_Br was anchored to a carbon cloth (**CC**) electrode surface via the formation of C−C bonds. The presence of the aniline moiety on the bipyridyl ligand enables the grafting of the complex onto carbon surfaces via the in situ formation of the corresponding diazonium salt. This method advantageously bypasses the isolation and purification of the diazonium reactive intermediate. Electrochemical reduction is performed to trigger C−C bond formation and N_2_ evolution. To assess the amount of electrocatalyst covalently bound on **CC**, a comparative ICP analysis was performed on the pristine **CC** starting material and on **Mn/CC**. A surface coverage Γ of 1.67×10^−9^ mol cm_ECSA_^−2^ was obtained, in reasonable agreement with the previously reported value (1.4×10^−9^ mol cm_ECSA_^−2^), determined via the indirect method of charge integration of the CV data collected from **CC** bonded nitroaniline.^[23]^ XPS measurement was performed to further assess the state of the **Mn/CC** catalyst. The spectrum obtained from the catalyst sample before exhaustive electrolysis displays two peaks at 641.9 and 653.0 eV (Figure S2), which can be assigned to Mn(I) 2p3/2 and 2p1/2, respectively. This is in perfect agreement with previous studies on Mn(I) derivative that reported the two peaks at 641.8 and 653.0 eV.^[28]^

The modified **CC** electrode was first tested for CO_2_ electroreduction using a three‐electrode cell with two gastight compartments filled with methanol, kept under a constant flow of CO_2_ (30 mL/min). An onset reduction potential of –1.35 V vs. Ag/AgCl was applied.

With the exact same setup, CO_2_ reduction was performed with the **Mn/CC** modified electrode immersed into CO_2_‐saturated MeOH solution of each amine (1 mM) within the cathodic compartment. The different amines tested (Figure 2) were Diethylenetriamine (**DETA**), Diethanolamine (**DEA**), Pentamethyldiethylentriamine (**PMDETA**), Triethylamine (**TEA**), Tetramethyletylendiamine (**TMEDA**) and 2‐tert‐butyltetramethylguanidine (**TBG**).

**Scheme 2:**
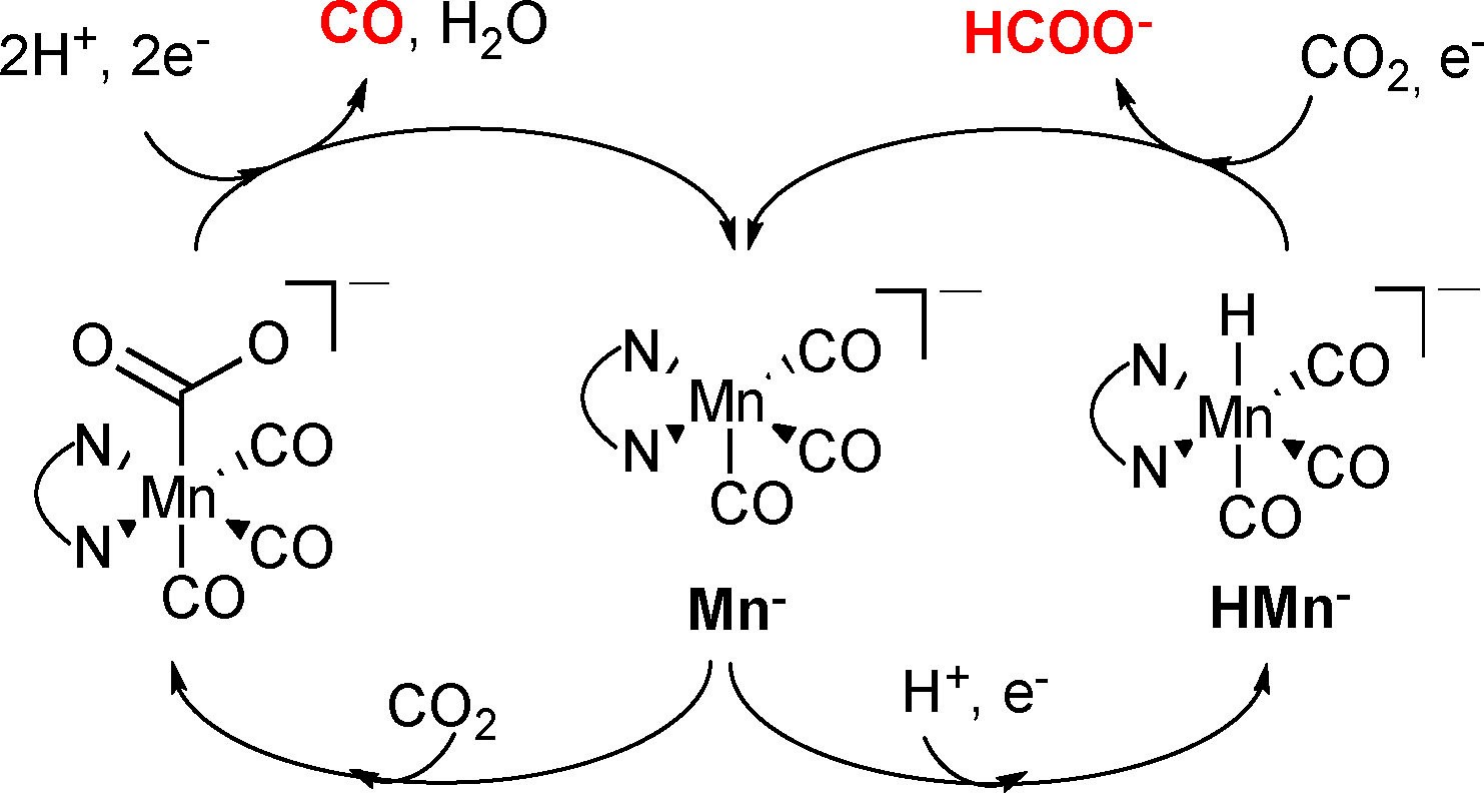
Schematic mechanism of CO_2_ reduction by Mn catalysts.

At such low amine concentration (1 mM), at least three orders of magnitude lower than what is commonly used for CO_2_ capture (MEA 5 M, 30 % w/w), most of the captured CO_2_ is absorbed by physical dissolution (see Table 1). In fact, at room temperature and under a partial pressure p(CO_2_)=1 atm, this gas has a solubility of 0.007 in MeOH,^[29]^ which translates into a concentration around 175 mM. Although our amine solutions are rather diluted (1 mM), the values of carbamation and carbonation equilibrium constants (Scheme 1) suggest that these processes remain quantitative in our operational conditions (see Supporting Information section 2 for the relationship between binding constant per nitrogen site and per absorbent molecule). This was experimentally verified by ^1^H NMR for **DEA**, **DETA** and **PMDETA**, which cover the scope of primary to tertiary amines, bearing between one and three binding sites and either undergoing preferentially carbamation or carbonation (see Supporting Information section 4). As Gibb's free energy of carbonation is lower compared to carbamation, we herein used a 50‐fold excess of water with respect to the amine, which enables the former process to be as favored as the latter (Table 1 and Scheme 1, Equation (2)). In our operating conditions, amines are stoichiometrically loaded with CO_2_. Yet, these adducts cannot be realistically considered as substrates for CO_2_ conversion, as they are 100 times less abundant than dissolved CO_2_. In addition, as reported in Table 1, they are also substantially more stabilized than the dissolved gas. In such conditions, the scenario wherein ammonium carbamates and carbonates (more stable and less abundant than dissolved CO_2_) act as substrates during electroreduction can eventually be ruled out, allowing us to focus on the potential role of these CO_2_‐amine derivatives in the very catalytic process. We have recently shown that industrial polyamines used for CO_2_ capture are powerful metal chelators which can effectively be employed for metal extraction in methanolic medium. In agreement with this previous study, we observed that 20 minutes of CO_2_ flow were required to fully pre‐load the amine solution and further enable the electrolysis to proceed properly. Any attempt to directly contact the unloaded diluted amine and the catalyst, systematically resulted in a detrimental effect on the reduction activity. In addition, DFT calculations also suggests that the real catalyst (i. e. the Mn pentacoordinate anion [Mn(bpy)(CO)_3_]^−^, **Mn^−^**, see Scheme 2 below) prefers to coordinate the free amine rather than CO_2_ or MeOH (see Supporting Information section 4). After the preliminary CO_2_ saturation, **Mn/CC** was inserted in solution, continuously supplied with CO_2_ while the electrolysis was conducted with a set potential of −1.35 V vs. Ag/AgCl. Karl‐Fischer titrations were performed at the beginning and at the end of the process, confirming that a constant amount of H_2_O was present in the medium, around 0.96 mg/mL (i. e. 0.1 % w/w or 50 mM).

**Table 1:** Gibb's free enthalpies of CO_2_ in different states and concentrations used in the present study.

State	Δ*G*° [kJ⋅mol^−1^]	Conc. [mM]
atm		0.018
flue		5.4
pure gas	+9.0	45
dissolved^[a]^	−12.9	175
bicarbonate^[b]^	−28.6	<3
carbamate^[b]^	−36.4	<1.5

[a] Calculated from CO_2_ solubility in MeOH. [b] From MEA in water (from Ref. [8]).

Table 2 shows the TONs (Turnover Numbers) and FEs (Faradic Efficiencies) obtained for all the different conditions tested. TON_CO_, TON_H2_ and corresponding FE values were obtained by sampling gases from the cell headspace every 5 minutes and by injecting them in a micro‐GC analyzer, while TON_HCOO−_ and FE_HCOO−_ were evaluated by quantitative ^1^H NMR analysis of the catholyte at the end of electrolysis.

**Table 2:** TONs and FE values for CO_2_ reduction with the **Mn/CC** electrode in MeOH (TBAPF_6_ 0.1 M as supporting electrolyte) with and without amines (1 mM).

Diluent	Amine (1 mM)	Time [h]	TON_CO_	TON_H2_	TON_HCOO−_	FE_CO_ [%]	FE_H2_ [%]	FE_HCOO−_ [%]
water	–^[a]^	10	33200	28800	0	60	40	0
methanol	–	22	10360	3900	5150	51	20	26
	DETA	22	7960	3860	16160	26	12	51
	DEA	20	8530	6230	7780	29	21	25
	PMDETA	22	3000	6700	28000	5	11	66
	TEA	21	5735	6740	4040	20	23	15
	TBG	22	6159	5613	6563	21	19	22
	TMEDA	15	10073	16771	873	26	44	3

[a] Water solution with no added amines (Ref. [23a])

**Figure 3:**
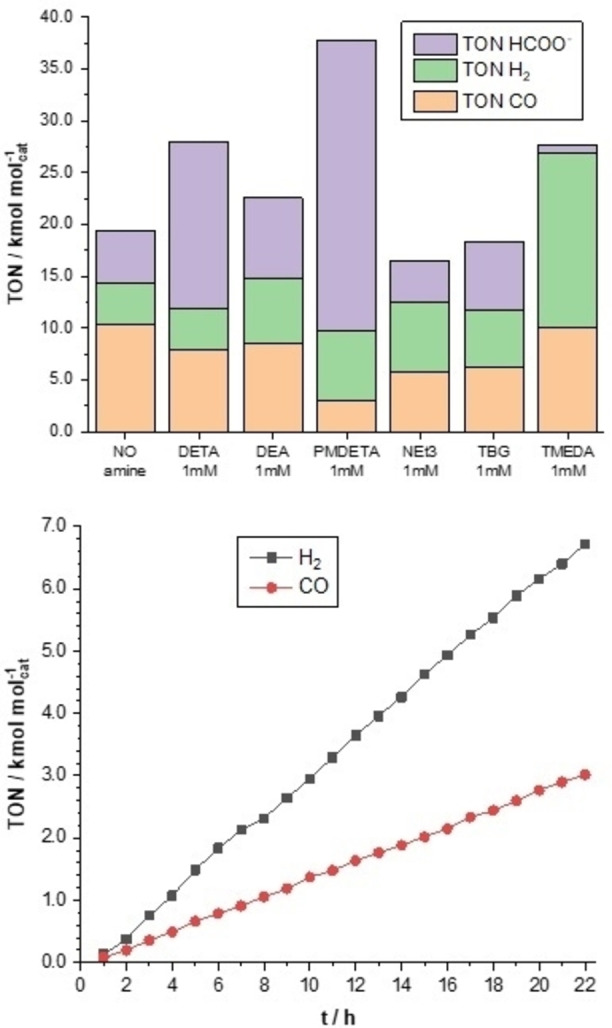
Overall efficiencies after 22 h for the amines represented in Figure 2 (top). TON time profile for **Mn/CC** with **PMDETA** under continuous flow of CO_2_ (bottom).

Figure 3 displays the overall TON_H2_, TON_CO_ and TON_HCOO−_ values after 22 h of continuous CPE at −1.35 V for all amines (E_red_=−1.35 V, TBAPF_6_ 0.1 M), and the production of H_2_ and CO over time of a methanolic solution containing **PMDETA**.

Some general observation can be made from the results gathered in Figure 3 and Table 2. We previously reported that the same **Mn/CC** catalyst only afforded CO (FE_CO_=60 %) and H_2_ (FE_H2_=40 %) as CO_2_ reduction products when used in aqueous medium,^[23a]^ while CPE in MeOH displays FE_CO_=51 %, Fe_H2_=20 % and FE_HCOO−_=26 % (Table 2). It is now accepted that the production of formate occurs via the formation of the hydride **HMn** intermediate, while CO_2_ reaction with the active catalysts **Mn** ^−^, followed by the protonation‐first or reduction‐first mechanisms lead to CO (Scheme 2).[^18b^, ^20^, ^30^]

Clearly, by simply switching from aqueous to methanolic solutions, a change in reduction selectivity occurs. The same shift in selectivity in favor of formate (path via **HMn^−^** in Scheme 2) may be obtained in water by using gas diffusion layer (GDL) electrochemical cell, resulting into FE_CO_=76.2 %, FE_H2_=13.7 % and FE_HCOO−_=10.1 %.^[23b]^ The effect has been ascribed to acidification induced by increased CO_2_ concentrations, symptomatic of GDL cells. Following this track, we tried to further shed light on the chemical process leading to formate production in methanol. This may either be imputed to higher CO_2_ concentration (as in water^[23]^) and, when any, to a non‐innocent‐role played by the amine in the catalytic reduction mechanism.

Within the series tested, **DETA** induced a significant increase in CO_2_ selectivity to formate, reaching high FE_HCOO−_ of 51 %, while **DEA** had no significant effect on the catalyst's activity or selectivity (FE_HCOO−_=25 %). The most striking results were obtained by the addition of **PMDETA**, which strongly shifted the selectivity of **Mn/CC** towards formate, with a remarkable FE_HCOO−_ of 66 %. Noteworthy, while FE_H2_ of **DETA**, **DEA** and **PMDETA** are similar (12, 21 and 11 %, respectively), FE_CO_ values of **PMDETA** is significantly lower (5 %). The main difference between the three amines is that **PMDETA** presents three tertiary amine functionalities, which orients CO_2_ capture exclusively toward carbonation (Eq. (2), Scheme 1). For this reason, three other tertiary amines or guanidine were tested at the very same concentration: **TEA**, **TMEDA** and **TBG**. These species respectively present one, two and three tertiary amine functionalities, which are conjugated into a guanidine pattern in the latter. Surprisingly, these additives did not induce an increase in the production of formate compared to the amine‐free reference system. In contrary, **TMEDA** displayed an unexpected detrimental effect on formate production, yielding TON_CO_ values similar to those obtained in the absence of amine, and a noticeable increase in FE_H2_ and TON_H2_. **TEA**, **TMEDA** and **TBG** reached TON_HCOO−_ values of 4040, 873 and 6563, and TON_CO_ of 5735, 10073 and 6159, respectively. From this set, it appears that **PMDETA** was the most efficient catalytic additive, favoring the reduction of CO_2_ into formate in methanolic solutions with high TON and FE.

Daasbjerg and coworkers^[31]^ demonstrated that, in homogeneous conditions, the active catalytic species [Mn(bpy)(CO)_3_]^−^ (**Mn^−^**) reacts in acetonitrile with the starting neutral complex [Mn(bpy)(CO)_3_Br] (**Mn**) producing directly the neutral dimer, a key intermediate in the electrochemical reduction of the Mn bipyridyl complexes. We hypothesized that in the present case, MeOH can transform [Mn(bpy)(CO)_3_]^−^ into the corresponding hydride HMn(bpy)(CO)_3_, namely **HMn**, since the Mn catalyst is locked on the **CC** surface and it is unlikely to react with another Mn unit.

Selected DFT calculations, performed to explain the main trends and elucidate the underpinning mechanisms, clearly indicate that the proton of MeOH points towards the metal center of the pentacoordinated [Mn(bpy)(CO)_3_]^−^ complex, and that the chemical reaction [Mn(bpy)(CO)_3_]^−^+CO_2_+MeOH→[HMn(bpy)(CO)_3_]+MeCO_3_^−^ displays a favorable ΔG=−55.0 KJ/mol (see Supporting Information section 4).

**Figure 4:**
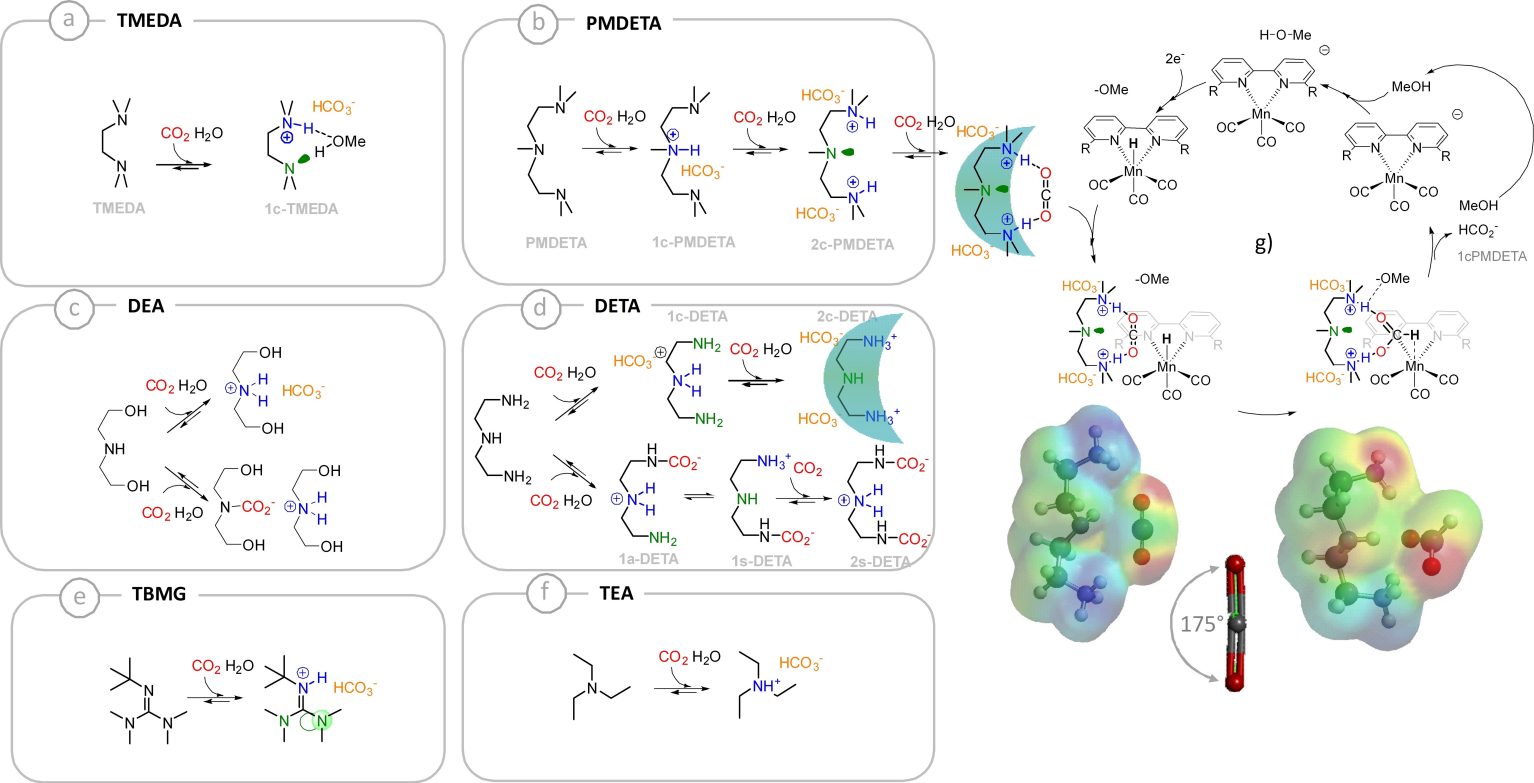
Interpretation of the selectivity observed from the set of amines used in this work. Frames a)‐f) display the members of the carbamate and carbonate libraries generated upon CO_2_ capture by each absorbent at 1 mM in methanolic solutions containing 50 mM of water. The green‐cyan arch symbolizes the quadrupolar profile of bis‐ammonium **2 c‐PMDETA**, which displays electrostatic complementarity for CO_2_ and activates its reaction with the hydride generated from methanol on the supported Mn catalysts.

NMR analyses performed on amine samples at different concentration in CD_3_OD but with a fixed 50 mM D_2_O provided some clues about the species that may be present in the cathodic compartment and on their relative abundance (Supporting Information section 3). On average, amines can be loaded with around 0.3–0.4 equiv. of CO_2_ per nitrogen site at 500 mM. This loading does not vary substantially upon dilution with CO_2_‐saturated CD_3_OD containing 50 mM D_2_O, as attested by measurement of the protonation state (from ^1^H chemical shift and potentiometry) and by the absence of stripping. For amines bearing primary and secondary nucleophilic nitrogen binding sites such as **DEA** and **DETA**, dilution from 500 to 5 mM globally switches the CO_2_ fixation pathway, from carbamation to carbonation. At high alkalinity/amine concentration, methyl carbonate MeCO_3_^−^ is observed as a carbonation side product. For **PMDETA**, a 1.05 : 1.00 HCO_3_^−^: amine molar ratio is obtained (see Supporting Information), which validates the formation of catalytic amounts of biprotonated **PMDETA**, named **2 c‐PMDETA** (see Figure 4 and discussion below). At the working potential of −1.35 V, the hydride complex **HMn** is reduced to its corresponding electron‐rich radical anion **HMn^−^**, which is the real catalyst for CO_2_ to formate conversion. DFT calculations indicate that the irreversible reduction potential of **HMn** is less negative by ∼65 mV than that of the corresponding Mn dimer.

By using **Mn^−^** as model, DFT calculation performed at high level def2‐TZVP basis set allowed us to elucidate the two mechanisms depicted in Scheme 2, in MeOH as solvent. The path leading to CO passes through the coordination of the weak electrophile CO_2_ to the strong nucleophile **Mn^−^** (described in the Supporting Information), whereas the formate production in MeOH proceed via the hydride **HMn** and its reduced form **HMn^−^**. Table 3 summarizes the relevant intermediates and Transition States found for this system. The first step consists into the weak coordination of CO_2_ to the **HMn^−^** radical anion. The adduct (**Mn‐H^−^⋅⋅⋅CO_2_**) produces the intermediate (**Mn⋅⋅⋅H‐CO_2_** ^**−**^), which is 57.4 KJ/mol more stable than the (**Mn‐H^−^⋅⋅⋅CO_2_**) precursor, via the transition state **(Mn⋅⋅⋅H^−^⋅⋅⋅CO_2_)^TS^**. The energy barrier is only 11.0 KJ/mol. Thus, in MeOH, formate coordinates to the metal preferentially by its hydrogen rather than its oxygen atom, at least as a first step. Subsequently, the complex rearranges, passing through another transition state in which the formate rotates: the energy of (**Mn⋅⋅⋅H‐CO_2_** ^**−**^)^**TS**^ is only 2.9 KJ/mol higher than the intermediate (**Mn⋅⋅⋅H‐CO_2_** ^**−**^), (54.5 KJ/mol lower in energy with respect the starting (**Mn‐H^−^⋅⋅⋅CO_2_**) adduct). The final formate complex (**Mn⋅⋅⋅OCHO^−^**) is more stable than the starting species by 78.4 KJ/mol. Subsequent release of formate anion and electron transfer restores the starting radical anion catalyst **Mn^−^**. The mechanism leading to the coordination of CO_2_ to **Mn^−^** (and CO production) displays a similar energy barrier (10.0 KJ/mol, see Supporting Information section 4).

**Table 3:** Computed structures and relative energies (in KJ/mol) for the mechanism leading to formate. All the species are radical anions. Bottom row depicts reactant and product of the barrierless reaction **2 c‐PMDETA**+CO_2_+**HMn^−^** →**2 c‐PMDETA**+**[CO_2_HMn]^−^**.

Energy	Name	Structure
0.00	(Mn‐H^−^⋅⋅⋅CO_2_) (HMn^−^+CO_2_)	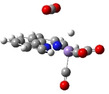
11.0	(Mn⋅⋅⋅H^−^⋅⋅⋅CO_2_)^TS^ TS: −448 cm^−1^	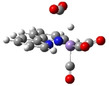
‐57.4	intermediate (Mn⋅⋅⋅H‐CO_2_^−^)	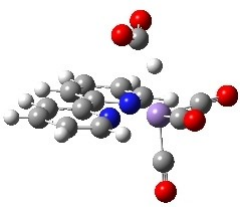
‐54.5	(Mn H‐CO_2_^−^)^TS^ TS: −30 cm^−1^	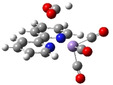
‐78.4	formate complex (Mn⋅⋅⋅OCHO^−^)	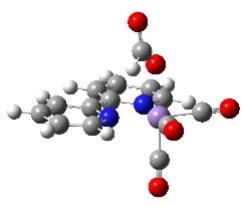
	reactant	product
	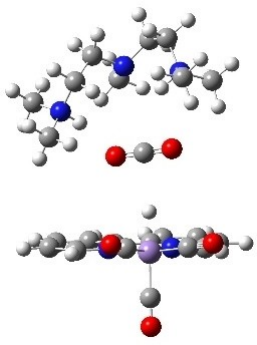	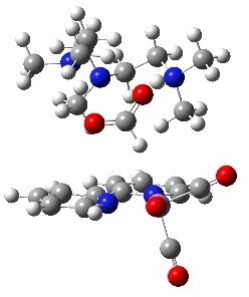

As mentioned earlier, adducts generated from diluted amine‐CO_2_ solutions (Figures 4 a‐f) should take part in this catalytic scenario, by either activating some reactive species (such as dissolved CO_2_) and/or by stabilizing key transition states. With the exception of **TMEDA**, all amines display a similar TON_H2_. This strongly suggests that the main source of protons in solution is MeOH rather than the ammonium moieties paired with carbamates or carbonates.[^19c^, ^20^]

**TMEDA** stands as an extreme case in the series, markedly favoring H_2_ over formate. The conversion of this α,β‐diamine into a bisammonium dication upon double carbonation is strongly disfavored, therefore its main adduct with CO_2_ combines a basic nitrogen moiety and an ammonium group (species **1 c‐TMEDA**, Figure 4a). This Lewis acid‐base pair (or dipole) can activate the reactivity of the MeOH dipolar species toward **HMn**, thereby leading to an increased H_2_ production. **PMDETA**, which formally results from the chain elongation of **TMEDA**, should act similarly. Yet, dicarbonation does occurs in substantial amount on this species, yielding an α,ω‐bisammonium bearing a central neutral nitrogen (Figure 4b, **2 c‐PMDETA** and Supporting Information section 3). This quadrupolar species perfectly meets the requirements to activate a complementary quadrupolar species, such as CO_2_ (Figures 4b and g). Preliminary calculations at the AM1 level (Figure 4g, bottom) confirm this electrostatic complementarity and affinity (with the starting material, dissolved CO_2_, and the product, the formate anion). It also highlights the activating role of **PMDETA**, as CO_2_ binding is accompanied by its bending by 5°, which pre‐activates this substrate toward hydride addition (figure 4g, bottom).

Additional DFT calculations confirm this interpretation. The divalent ammonium‐bicarbonate salt **2 c‐PMDETA** does not only bind quite strongly to CO_2_ (computed ΔG=−9.7 KJ/mol), but the reaction with the reduced Mn hydride radical anion **HMn^−^** proceeds barrierless toward the production of formate, which is coordinated to Mn metal via the H atom (see last row in Table 3). The catalytic cycle then proceeds as previously described and illustrated in Table 3. Thus, the limiting step of the whole catalytic cycle is apparently no longer CO_2_ activation, but the concentration in solution of the adduct **2 c‐PMDETA ⋅ CO_2_**. Its concentration is limited by many equilibria present in the solution that compete with CO_2_ for the coordination of **2 c‐PMDETA** (i. e. bicarbonate, formate, methyl carbonate, methanol and water).

The model described in this paragraph, based on the ability of absorbents to generate dipolar or quadrupolar CO_2_ capture adducts in significant amounts, which should respectively activate complementary dipoles or quadrupoles such as methanol or CO_2_ and enhance the production of H_2_ or formate, remains valid on the rest of the series. **DETA** stands in between the two extreme cases of **PMDETA** and **TMEDA** and moderately enhances the selectivity toward formate production. We recently reported that **DETA**‐CO_2_ is a compositionally complex system in methanol^[3d]^ which is herein even further complexified by the presence of water (Figure 4c). While some methanol activating dipolar patterns can be found on some members of the **DETA**‐based library of carbamates and carbonates (such as species **1 c‐DETA**, **1 a‐DETA** and **1 s‐DETA**, Figure 4d), the presence of appended charges or polar moieties seems to prevent any enhancement of H_2_ production. The bisammonium biscarbonate homologue of the **2 c‐PMDETA** adduct, noted **2 c‐DETA** is present in this complex system, but its relatively lower concentration and higher hydrophilicity (which decreases the availability of the ammonium moieties for lone pair binding) moderates the enhancing effect toward formate production. The other amines of the series behave as negative controls (Figures 4c, e and f): triethylammonium only displays a single acidic site while the free doublets on **TBG** are orthogonal to the lone pair of the only free nitrogen site. **DEA** bears two alcohol end groups, which may act as moderate H‐bond donating sites, but in its loaded form, it misses the central nucleophilic nitrogen, to properly play the co‐factor role imputed to **2 c‐PMDETA** quadrupole.

## Conclusion

To the best of our knowledge, this study proposes the first example of a covalently bound organometallic complex devoted to CO_2_ reduction in the presence of industrial amines in wet methanolic medium. Compared to previous integrated CO_2_ capture and electroreduction processes, the current system opens the possibility of scaling up the entire carbon capture and recycling chain, by employing a cost‐effective Mn‐based electrocatalyst. The abrupt and on‐demand increase of FE_HCOO−_ and TON_HCOO−_ values obtained by using **PMDETA** as additive represents a breakthrough in the catalytic activation of CO_2_. Besides the technological perspectives, this study provides valuable knowledge on the potential synergies occurring at the molecular level between capture agents and supported catalysts. Combined experimental and theoretical approach allowed us to elucidate the role of the absorbent (an effector or inhibitor) and the diluent (proton source and CO_2_ reservoir). In silico DFT studies elucidated the entire mechanism of the process, which was confirmed to diverge on demand toward CO or formate production depending on the structure of the amine additive used. In particular, an unexpected barrierless conversion of CO_2_ to formate was observed in our operating conditions with **PMDETA**. A lock‐and‐key scenario emerges from this analysis to explain the role of CO_2_‐loaded amines in the activation of either dipolar (methanol) or quadrupolar (CO_2_) substrates towards the reaction with the Mn catalyst, enabling to tune the reaction toward H_2_ or formate production.

We believe that this study should stimulate many investigations on the synergies between CO_2_ capture and conversion by electrochemical and chemical means. It clearly calls for further improvement, such as increasing the effective concentration of CO_2_ and facilitating the release of formate. Yet, it brings the proof of feasibility of amine‐assisted enhancement of electrochemical activity and selectivity, on a catalytic systems fulfilling several of the many requirements for potential industrial deployment.

## Experimental Section

### General considerations

CV and CPE experiments were performed using an Amel 7050 potentiostat. Gaseous reduction products (CO and H_2_) were detected and quantified with an Agilent 490 μGC with two separate CP‐Molsieve 5 Å columns, equipped with a thermal conductivity detector. Columns were kept at 85 °C and at a pressure of 21 psi. CO and H_2_ were quantified using He and Ar as carrier gases, respectively. The gas inside the measurement cell was sampled for 30 s every 5 min to fill the μGC 10 μL sample loop, and eventually 400 nL were injected into the column. Ar, He, and CO_2_ pure gases (>99.9995 %) from Sapio have been used, and two different certified standard concentrations of CO and H_2_ in Ar matrix (Rivoira) for μGC calibration.^[17]^ Detection limits for CO and H_2_ were 1 ppmv and 0.5 ppmv, respectively. Formate was quantitatively detected by q^1^H NMR recorded on a JEOL ECP 400 FT NMR spectrometer (^1^H operating frequency 400 MHz) at 298 K using DMSO as an internal standard. ^1^H chemical shifts are reported relative to TMS (δ=0) and referenced against solvent residual peaks. All reagents and solvents were obtained from commercial sources at reagent‐grade purity and used as received. Diethylenetriamine (99 %), N,N,N’,N’‐Tetramethyl Ethylenediamine (∼99 %), N,N,N’,N’’,N’’‐Pentamethyldiethylenetriamine (99 %), Diethanolamine (≥98 %), Triethylamine (≥99.5 %), 2‐tert‐butyltetramethylguanidine (≥97 %) were purchased from Sigma‐Aldrich. CO_2_ (99,95 %) and N_2_ (≥99.9 %) were obtained from Air Liquid. D_2_O (99,90 % D) was purchased from Eurisotop.

### Electrochemistry

Electrochemical measurements were conducted in methanol (Sigma‐Aldrich, assay GLC≥99.9 %) with tetrabutylammonium hexafluorophosphate (TBAPF_6_ 0.1 M) as supporting electrolyte. TBAPF_6_ (Sigma‐Aldrich, 98 %) was recrystallized twice from hot ethanol and dried before use. A single compartment cell was used for CV measurements. The pristine carbon cloth, **CC**, (GPP050 M, Cetech Co. ltd) was cut in pieces of 3.4 cm^2^ and ultrasonically cleaned in 50 % wt 2‐propanol solution in Milliq water for 15’ and in pure Milliq water for additional 20’, then dried in an oven at 60 °C for one hour, in a similar manner as previously reported.^[23b]^ A double compartment cell was used for CPE measurements. **CC** was employed as working electrode, alongside a Pt counter electrode and an Ag/AgCl (KCl 3 M) as reference electrode. Under our experimental conditions, the reference ferrocene/ferrocenium (Fc/Fc^+^) redox is at E_1/2_=0.40 V (ΔE_p_=65 mV) vs. Ag/AgCl.

### General procedure for controlled potential electrolysis

Functionalized **Mn/CC** electrodes were tested for CO_2_ electroreduction in methanol, their performance was studied by means of Controlled Potential Electrolysis (CPE). TBAPF_6_ (0.1 M, 1.548 g) was dissolved in methanol (40 mL) in a three‐electrode cell with two gastight compartments under Ar. The gas flow was saturated with methanol vapors by a pre‐bubbler system in order to avoid evaporation in the cell.

Procedure with no addition of amines: the solution was saturated with a constant flow of CO_2_ (30 mL/min, 20 minutes). The electrolysis was started by setting a potential of −1.35 V vs. Ag/AgCl.

Procedure with addition of amines: specific aliquots of the amines (1 mM) were added in the cathodic compartment. The solution was saturated with a constant flow of CO_2_ (30 mL/min, 20 minutes) prior to insert **Mn/CC** electrode, in order to fully load the amine (this procedure is mandatory to avoid the direct interaction of the amine moieties with the catalyst, resulting in detrimental effect on the reduction activity). The electrolysis was started by setting a potential of −1.35 V vs. Ag/AgCl.

CPE was performed for 15–22 h during which the electrochemical cell was protected from light.

## Conflict of interest

The authors declare no conflict of interest.

1

## Supporting information

As a service to our authors and readers, this journal provides supporting information supplied by the authors. Such materials are peer reviewed and may be re‐organized for online delivery, but are not copy‐edited or typeset. Technical support issues arising from supporting information (other than missing files) should be addressed to the authors.

Supporting Information

## Data Availability

The data that support the findings of this study are openly available in ChemRxiv at https://doi.org/10.26434/chemrxiv‐2021‐j24z0, reference number 0.
