# Supplementary material for: Efficient Electrochemical Reduction of CO2 to Formate in Methanol Solutions by Mn‐Functionalized Electrodes in the Presence of Amines
Source: Chemistry. 2022 May 25;28(37):e202104377. doi: 10.1002/chem.202104377 (PMC9325359; doi:10.1002/chem.202104377)
Supplement: Supplementary file 1 — Supporting Information [file CHEM-28-0-s001.pdf]

Chemistry–A European Journal

Supporting Information

Efficient Electrochemical Reduction of CO_2 to Formate in Methanol Solutions by Mn-Functionalized Electrodes in the Presence of Amines

Francesca Marocco Stuardi, Arianna Tiozzo, Laura Rotundo, Julien Leclaire,*
Roberto Gobetto,* and Carlo Nervi*

Supporting Information

1. Preparation and characterization of the heterogeneous catalyst 1
2. Turn Over Numbers during CPE experiments.. 4
3. Thermodynamics of polyamine Carbamation in Methanol 5
4. NMR analyses of the amine-CO_2 systems in methanol 6
5. DFT Calculations 8
Supporting References 22

1. Preparation and characterization of the heterogeneous catalyst

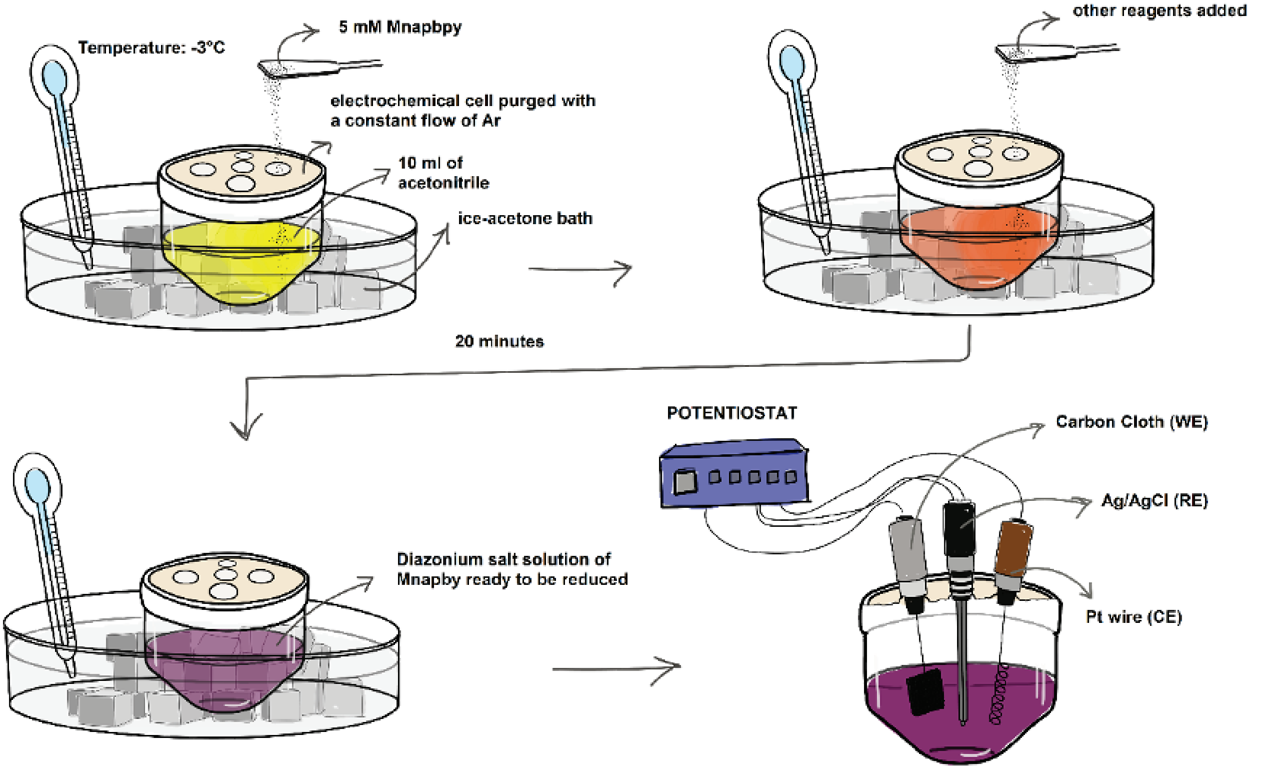

Figure S1. General procedure for **CC** functionalization.

Synthesis of *fac*-Mn(apbpy)$(CO)_3$Br

All commercially obtained reagents and solvents were used as received (Sigma-Aldrich). 4-(4-aminophenyl)-2,2'-bipyridine was synthetized according to published procedures.[1] [Mn(CO)5Br] (0.100 mmol, 1 equiv) and 4-(4-aminophenyl)-2,2'-bipyridine ligand (0.101 mmol, 1,01 equiv) were dissolved in sealed flasks containing 5 mL of diethyl ether and heated in a Biotage Initiator microwave reactor. The reaction mixture

was heated at 75°C for 15 min by high-frequency microwaves (2.45 GHz). After cooling to room temperature, the mixture was centrifuged and washed three times with diethyl ether. The solid sample was dried with a moderate flow of Ar as inert gas.

General Procedure for Electrode Functionalization

Pristine **CC** was treated as previously described. For the functionalization, *fac*-Mn(apbpy)$(CO)_3$Br (5 mM, 0.0233 g) was dissolved in acetonitrile (10 mL) under Ar atmosphere in a single-compartment electrochemical cell immersed in an ice-acetone bath (temperature under 0°C). Trichloroacetic acid (TCA) (30 µL) and isoamyl nitrite (20 µL) were added to the solution: the pale-yellow solution became orange due to the *in situ* generation of the diazonium salt. The solution was stirred for 20 minutes, then $TBAPF_6$ (0.1 M, 0.387 g) was added as supporting electrolyte and the electrodes were immersed in solution. Five CVs scans towards negative potentials were performed to reduce the diazonium salt and to induce the C-C bond formation between the complex and the carbonaceous surface of the support. After the functionalization, **Mn/CC** was sonicated in CH_3CN for 10 minutes and dried under inert gas (Ar).[1b]

The quantity of complex chemically bonded onto the **CC** electrode was evaluated by a comparative ICP analysis (Optima 7000 DV Perkin Elmer) on the pristine **CC** material and on **Mn/CC**. The samples were treated with a mixture of nitric acid and hydrogen peroxide to hydrolyze the C-C bonds. Two replicates were carried out for blank and sample and the concentrations found were: blank (pristine **CC** material) 0.083 ± 0.007 mg/kg and sample (**Mn/CC**) 49 ± 2 mg/kg. Considering that the sample weight was 0.0374 g, the catalyst loading on the functionalized carbon cloth is 3.34×10^{-8} mol. The real electrochemical active surface area (ECSA) was estimated by performing cyclic voltammetry on a 1.0 mM solution of ferrocene (Fc) at different scan rates (the diffusion coefficient of Fc is 2.24×10^{-5} cm^2 s^{-1}). The peak current was then plotted against the square of the scan rate and the slope is used to determine the real surface area according to the Randles-Sevčik law. The estimated roughness factor (fr = A_{ECSA}/A_{GEOM}) was 5.87, in agreement with the data previously reported by us.[1b] A CC with geometric area of 3.4 cm^2 has an ECSA of 19.95 cm^2, and hence a surface coverage of 1.67×10^{-9} mol $cm^{-2}{}_{ECSA}$.

XPS Characterization.

XPS spectra of **Mn/CC** were recorded before and after 22 hours of continuous electrochemical reduction in the presence of **PMDETA**, in the conditions outlined in Figure 3 (see main text). The Mn 2p spectrum of the sample before exhaustive electrolysis displays two peaks at 641.9 and 653.0 eV (Figure S2), which can be assigned to Mn(I) 2p3/2 and 2p1/2, respectively, in perfect agreement with previous studies on Mn(I) derivative that reported the two peaks at 641.8 and 653.0 eV.[2] It is important to note that the Mn complex is distributed into a single molecular layer over an irregular carbon cloth surface. Such a repartition delivers weak signals which are close to the XPS detection limit. During our past experience, we have noticed that the **Mn/CC** electrode continues to work as electrocatalyst until a negative potential is applied. When the experiment is interrupted, and **Mn/CC** exposed to the air, the catalyst undergoes decomposition, and its performance is significantly reduced (see ref 23). Thus, it is not surprising that XPS analysis performed on **Mn/CC** after exhaustive electrolysis (and after subsequent washing with methanol and water in order to remove the supporting electrolyte), delivers very noisy XPS signals in the same region (Figure S2). In order to confirm this interpretation, we also performed ICP-OES analysis on another carbon cloth electrode immediately after exhaustive electrolysis, but without washing it. The direct digestion of the exhausted **Mn/CC** by nitric acid and hydrogen peroxide mixture and titration of the Mn content in the leachate delivered a value of 1.02×10^{-9} mol $cm^{-2}{}_{ECSA}$ of Mn, which compares well with the 1.67×10^{-9} mol $cm^{-2}{}_{ECSA}$ value found on the starting **Mn/CC**.

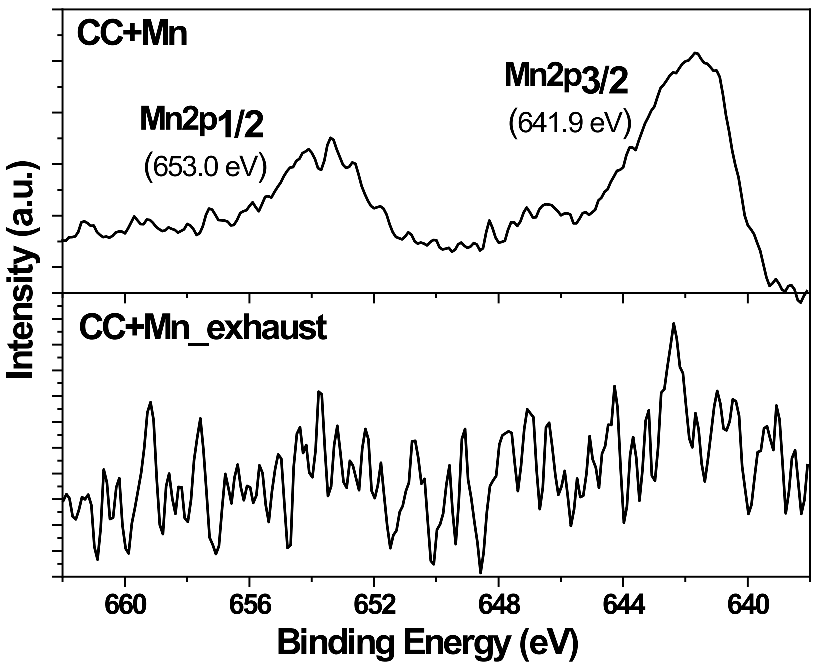

Figure S2. XPS spectra recorded on **Mn/CC** before (up) and after (down) CPE measurements.

A PHI 5000 Versaprobe Scanning X-ray Photoelectron Spectrometer (Physical Electronics, Chanhassen, MN, USA) with monochromatic Al K-alpha X-ray source (1486.6 eV energy), was used to investigate the chemical composition of the catalytic material. A spot size of 100 µm was selected to collect the photoelectron signal for both the high resolution (HR) and the survey spectra. Different pass energy values were exploited: 187.8 eV for survey spectra and 23.5 eV for HR peaks. All samples were analyzed with a combined electron and Ar ion gun neutralizer system, to reduce the charging effect during the measurements. The semi-quantitative atomic compositions were obtained using Multipak Version 9.7.0.1 dedicated software. All core-level peak energies were referenced to C1s peak at 284.5 eV.

2. Turn Over Numbers during CPE experiments

Figure S3 displays the time TON evolution for all the amine studied (**PMDETA** is reported in the main manuscript).

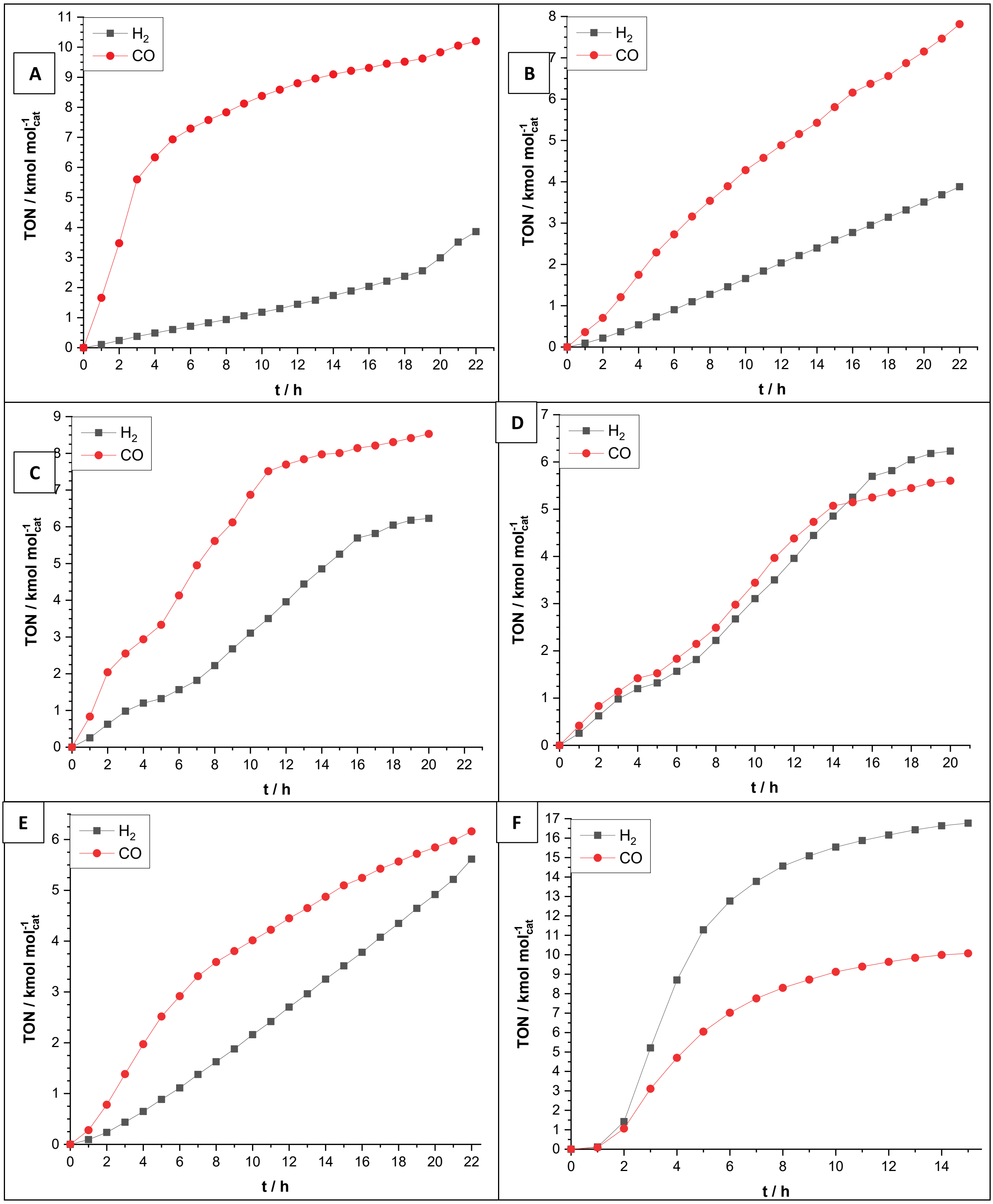

Figure S3. Turn Over Number (TON) time profile for **Mn/CC** with no addition of amine (A), with **DETA** (B), with **DEA** (C), with **NEt$_3$** (D), with **TBG** (E) and with **TMEDA** (F) under continuous flow of CO_2. Reduction potential –1.35 V in MeOH as solvent.

µGC calibration curves for CO and H_2 were obtained by using two different certified standards of CO and H_2 in Ar matrix. The first standard was CO 100.2 ppmmol, H_2 95.8 ppmmol and the second, CO 2001 ppmmol, H_2 2008 ppmmol. CO and H_2 were quantified by µGC analysis following the same procedure as previously reported.[3]

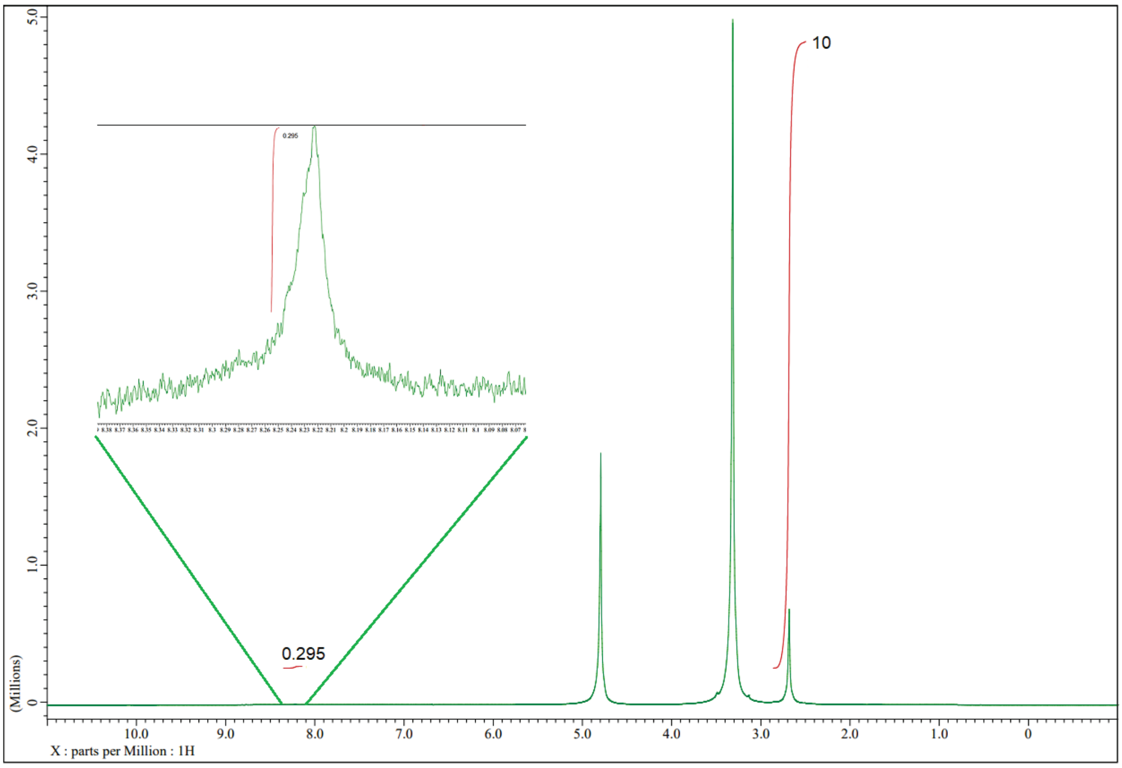

Figure S4. q¹H NMR recorded on a JEOL ECP 400 FT-NMR spectrometer for formate quantification in D_2O.

CPE experiment was performed in MeOH with 1 mM solution of **PMDETA** over **Mn/CC** electrode (see main text). After 22 hours of continuous CPE, 100 µL of solution was withdrawn from the cathodic compartment, added to 400 µL of D_2O and to 100 µL of an analytical grade DMSO solution as an internal standard (DMSO:D_2O 1:8). Formate was quantified by relative integration of the peaks. Figure S4 shows the whole q¹H NMR spectra, with the integrated DMSO and formate (in the inset) peaks.

3. Thermodynamics of polyamine Carbamation in Methanol

DETA + CO_2 = **1a-DETA** (4)

DETA + CO_2 = **1s-DETA** (5)

1s-DETA + **1a-DETA** + CO_2 = **1s:2s-(DETA)$_2$** (6)

3DETA + 4CO_2 = **1a-DETA + 1s:2s-(DETA)$_2$** (7)

which can be re-written as:

$$9R_1R_2NH + 4CO_2 = 4R_1R_2NH_2{+}\ 4R_1R_2NCO_2^- + R_1R_2NH \quad (7a)$$

and simplified into:

$$8R_1R_2NH + 4CO_2 = 4R_1R_2NH_2{+}\ 4R_1R_2NCO_2^- \quad (7b)$$

The corresponding equilibrium constants are related by the following equation:

$$K_7 = K_4*K_5*K_6 = K_{7b} = (K_1^{DETA})^4$$

With the previously measure constant values:

$$K_4 = 47.5\ M^{-1}, \qquad K_5 = 6.1\ M^{-1}, \qquad K_6 = 276.6\ M^{-1},$$

Leads to:

$$K_7 = 8.01\ 10^4\ M^{-4} \text{ and } K_1^{DETA} = 16.8\ M^{-1}$$

$$K_2^{PMDETA} = \frac{[HCO_3^-][R_1R_2NH_2^+]c°}{[CO_2][H_2O][R_1R_2NH]}$$

With c_0 c_0' and c defined as the initial concentration in amine group, water and CO_2 (both considered constant) and x the conversion factor, this transforms into:

$$K_2^{PMDETA} = \frac{(c_0x)^2 p°c°}{c\ c_0'\ c_0\ (1-x)}$$

and delivers:

$$K_2^{PMDETA} = 10.7$$

4. NMR analyses of the amine-CO_2 systems in methanol

A stock solution of amine (500 mM concentration) was prepared in CD_3OD. The solution was loaded with CO_2 by fluxing the gas at a constant flow rate (5 ml/min, 40 minutes). A precise aliquot of the solution was transferred in an NMR tube containing an internal reference (20 μl TMPCl 1 M in CD_3OD) and it was analyzed by quantitative ^{1}H and ^{13}C NMR. The stock solution was diluted 10 and 100 times with CD_3OD saturated with CO_2. The absence of stripping upon CD_3OD addition suggested CO_2 was not released upon dilution. The diluted samples were analyzed by quantitative ^{1}H NMR (20 μl TMPCl 1 M in CD_3OD).

The ^{13}C NMR spectrum of **PMDETA** (Figure S5) shows that CO_2 bubbling induces the formation of hydrogen and methyl carbonates in CD_3OD.

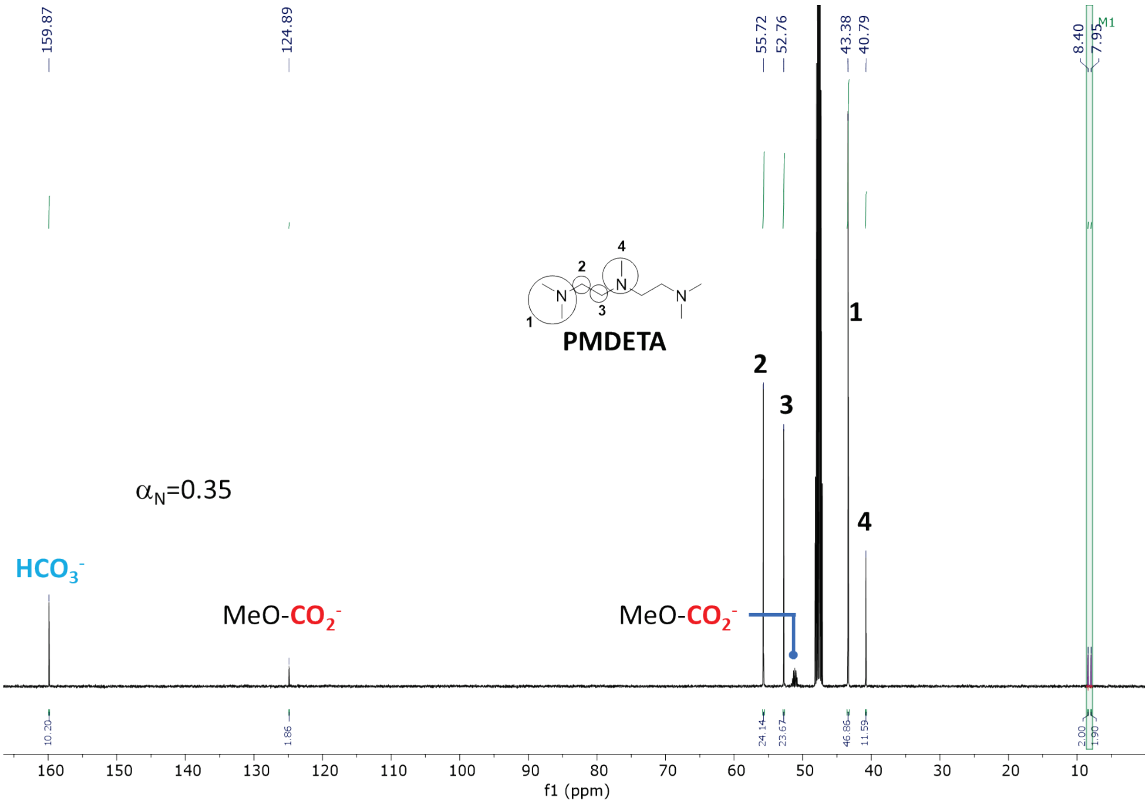

Figure S5. q^{13}C NMR spectrum of **PMDETA** (500 mM) in CD_3OD (TMPCl 0.04 M as internal reference). α_N= 0.35 indicates the loading per amine moiety and corresponds to a 1.05:1 HCO_3^-: **PMDETA** stoichiometry.

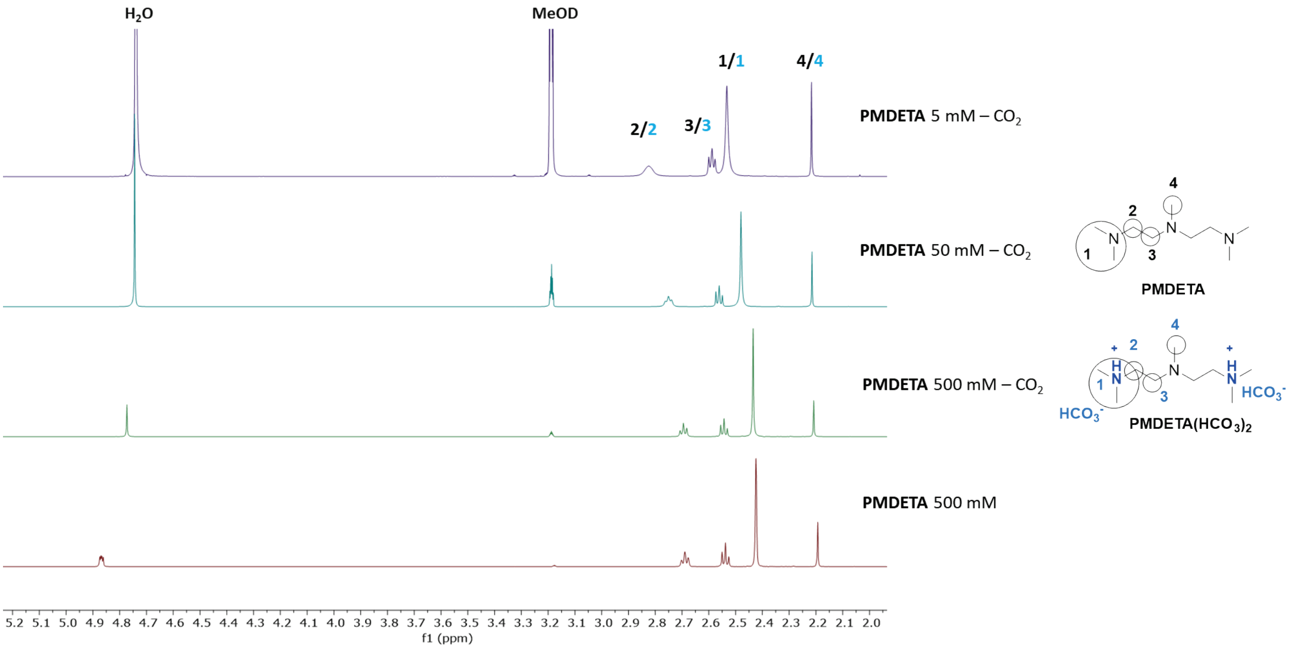

Figure S6. Stack of q^{1}H NMR spectra of **PMDETA**-CO_2 solutions in CD_3OD upon progressive dilution. Unloaded **PMDETA** (red) presented for comparison.

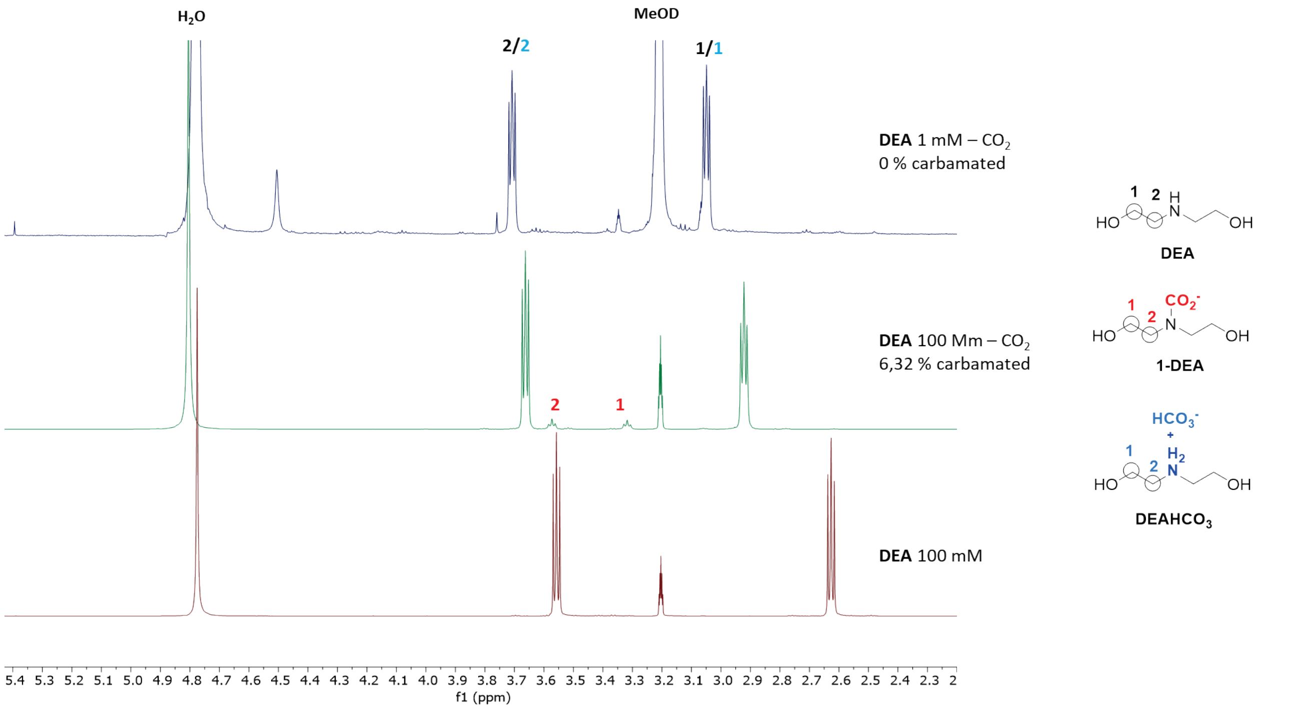

Figure S7. Stack of q^{1}H NMR spectra of **DEA**-CO_2 solutions in CD_3OD upon progressive dilution. Unloaded **DEA** (red) presented for comparison.

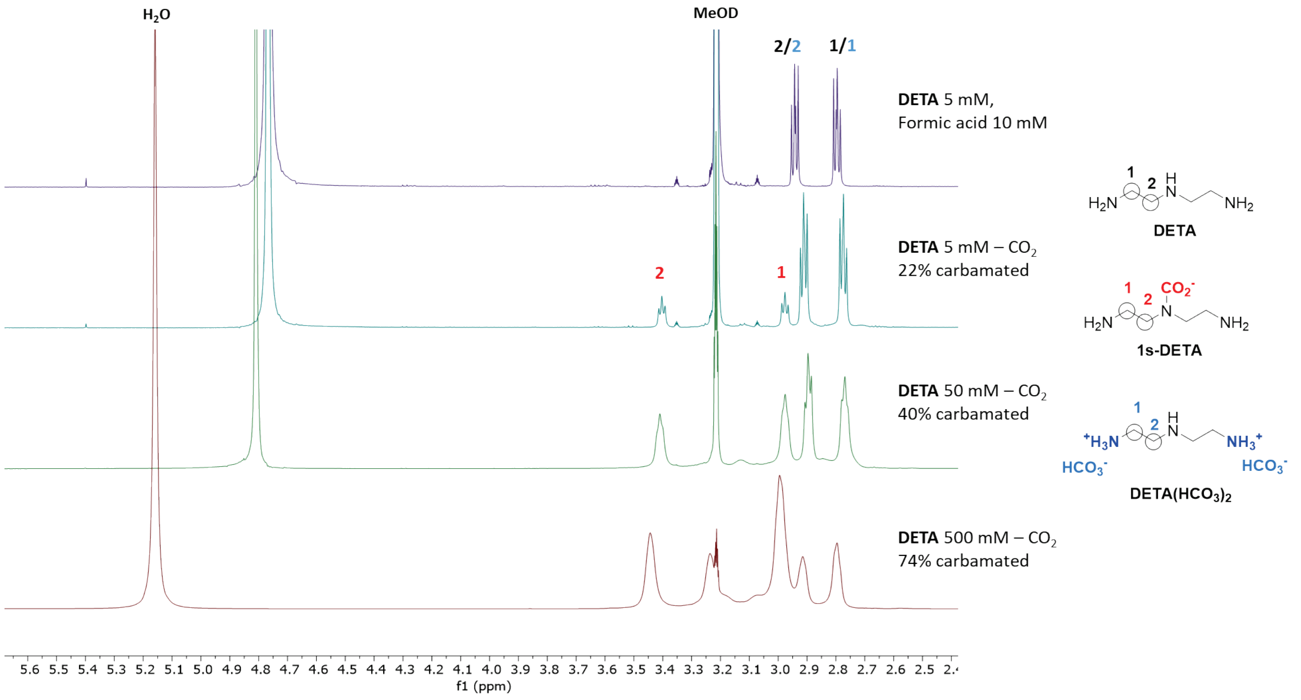

Figure S8. Stack of q^{1}H NMR spectra of **DETA**-CO_2 solutions in CD_3OD upon progressive dilution. Unloaded **DETA** (5 mM) + 2 equivalents of formic acid (10 mM) presented for comparison (purple spectrum).

5. DFT Calculations

DFT calculations were performed using Gaussian 09 Rev.D.01.[4] Solvent effects were included using the conductor-like polarizable continuum model (CPCM)[5] with methanol as solvent. Geometry optimizations were carried out without any constraints by a two-steps approach: the first by using the B3LYP functional,[6]

the optimized def2-TZVP basis set for Mn and Br, and the def2-SVP basis set[7] for all other atoms, while a second higher level and computationally demanding step has been performed using the triple ζ def2-TZVP basis set for all atoms. The D3 version of Grimme's dispersion method was applied adopting the Becke–Johnson damping scheme.[8] Zero point energies (ZPE) were routinely computed as correction to the electronic energies. For radical anions, unrestricted Kohn– Sham formalism (UKS) was adopted. The nature of all stationary points was confirmed by normal-mode analysis (no imaginary frequencies were found).

As evoked in the manuscript, we experimentally observed some **Mn/CC** catalyst degradation, whenever the functionalized electrodes were directly contacted with the unloaded amines, even in such diluted conditions. When preliminary CO_2 saturation was assessed, such a detrimental performance of **Mn/CC** catalyst was not observed. Based on our experience in combined CO_2 capture and metal extraction by polyamines, de-activation of the catalyst upon strong binding with the unloaded amine is quite likely. Furthermore, we performed DFT calculations with the aim of understanding the competitive coordination modes in the system containing $[Mn(bpy)(CO)_3]^-$ (**Mn**⁻) as catalyst model (the real catalysts responsible of CO_2 reduction), CO_2, methanol (MeOH) and the unloaded amine.

**Amine (1 mM)**	**CO_2 (KJ/mol)**	**MeOH (KJ/mol)**
**DETA**	13.4	26.5
**DEA**	27.4	41.6
**PMDETA**	29.6	22.5
**TEA**	21.3	17.3
**TBG**	9.4	26.5
**TMEDA**	17.9	6.6

Table S1. Stabilization energies (ZPE, in KJ/mol) when the amines are directly coordinated to Mn center instead of CO_2 and MeOH.

For each amine reported in Table S1, four structures were optimized: (**Mn**$^-$-CO_2)•(amine), (**Mn**$^-$-MeOH)•(amine), (**Mn**$^-$-amine)•(CO_2) and (**Mn**$^-$-amine)•(MeOH). The first two represent the direct coordination of CO_2 and MeOH to **Mn**$^-$ (with amine external, not directly coordinated to **Mn**$^-$), the latter two represent the direct coordination of the amines to **Mn**$^-$ with external CO_2 and MeOH, respectively. The energies values of columns **CO_2** and **MeOH** of Table S1 are thus relative to the following equilibria:

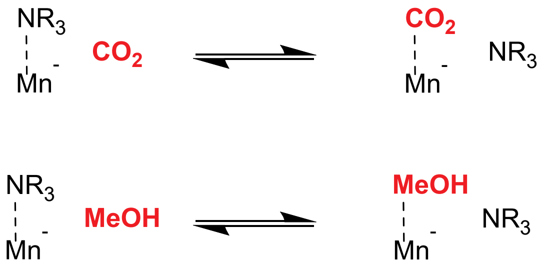

Scheme S1. Scheme of the equilibrium involving **Mn**$^-$.

In all cases **Mn**$^-$, namely $[Mn(bpy)(CO)_3]^-$, prefers to coordinate the amine rather than CO_2 or MeOH, as numerically shown by the ZPE positive values shown in Table S1.

The geometries of all the species were first obtained by calculations using the def2-TZVP basis set for Mn and def2-SVP for all other atoms. In a second step more accurate geometries and energies were obtained reoptimizing the structures with the def2-TZVP extended basis set for all atoms. By this approach a convenient speed-up in calculations was achieved. For Transition States (TS) the single negative frequency is reported.

Energies	Name	Structure
0	**$(Mn^{-}\cdots CO_2)$ (a)**	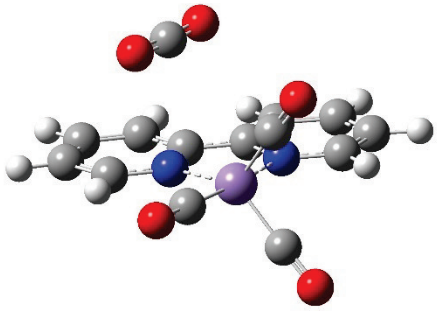
3.4	**$(Mn^{-}\cdots CO_2)$ (b)**	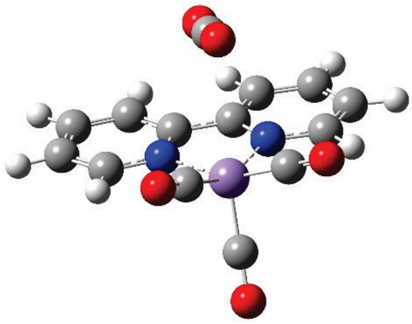
10.0	**$(Mn^{-}\cdots CO_2)^{TS}$**<br>TS: 265 cm^{-1}	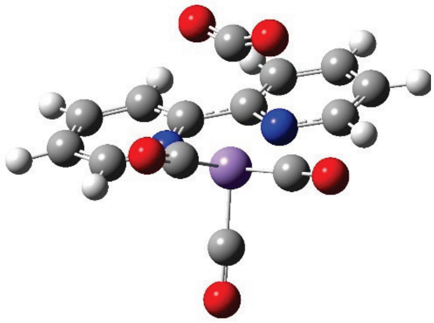
2.8	**$(Mn\text{-}COO^{-})$**	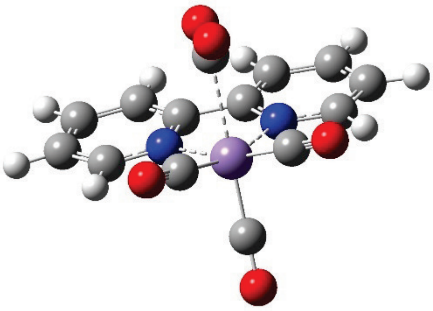

Table S2. Computed structures and relative energies (in KJ/mol) for the mechanism leading to the CO_2 coordination to the active catalyst **Mn**$^{-}$ (path leading to CO formation, see Scheme 2). All the species are anions.

The two mechanisms depicted in Scheme 2 were partially analyzed by DFT, employing MeOH as solvent. The production of formate has been analyzed in the manuscript, whereas the production of CO is briefly described here. The weak electrophile CO_2 tends to associate with the strong nucleophile **Mn⁻**. We identified two possible **(Mn⁻···CO₂)** adducts that display lower **(a)** and higher **(b)** symmetry (Table S3). They are very close in energies and O-C-O bond angles: 176.8° for **(a)** and 175.8° for **(b)**. The computed transition state **$(Mn^-\cdots CO_2)^{TS}$** is only 10.0 KJ/mol higher in energy, with a CO_2 bond angle of 153.0°. The bond angle of the coordinated CO_2, namely **(Mn-COO⁻)**, become 130.7°, but the energy is comparable with the starting adducts (see Table S2). Thus, a delicate equilibrium among all these forms can be established. In the absence of amines the energy barriers leading to CO or formate are more or less equivalent in MeOH, accounting for the balanced production. When **PMDETA** is used, the solution equilibrium with CO_2 and water produces **2c-PMDETA**, which shifts the selectivity towards the formate production via the **HMn** path because the barrierless activation described in the manuscript.

DFT calculations also indicate that the following equilibrium reaction is shifted to the right by 55.0 KJ/mol:

$Mn(bpy)(CO)_3]^- + CO_2 + MeOH \rightarrow [HMn(bpy)(CO)_3] + MeCO_3^-$

Similar equilibrium has been observed in NMR experiments, where **PMDETA**, although a weaker Brønsted base than $Mn(bpy)(CO)_3]^-$, plays the same role of proton acceptor.

An important point emerged from def2-SVP and def2-TZVP calculations: the def2-SVP, although being much faster than def2-TZVP, is qualitatively in agreement and can be used as rough estimation of the mechanism, whereas the def2-TZVP basis set is superior in term of accuracy. Table S3 demonstrates the good agreement between def2-SVP and the def2-TZVP calculations.

**Intermediate**	**Relative ZPE def2-SVP (kJ/mol)**	**Relative ZPE def2-TZVP (kJ/mol)**
**Mn^--H···CO_2**	0.0	0.0
**$(Mn\cdots H^-\cdots CO_2)^{TS}$**	11.1<br>TS: –425 cm^{-1}	11.0<br>TS: –448 cm^{-1}
**(Mn···H-CO_2^-)**	–28.9	–57.4
**$(Mn\ \ H\text{-}CO_2^-)^{TS}$**	–20.7<br>TS: –41 cm^{-1}	–54.5<br>TS: –30 cm^{-1}
(**Mn···OCHO⁻**)	–74.5	–78.4
**Mn^-** + CO_2 + MeOH ←→ **HMn** + $MeCO_3^-$	–24.4 KJ/mol	–55.0 KJ/mol

Table S3. Comparison of the relative energies between def2SVP and def2TZVP optimized structures.

Optimized structures (charge, spin multiplicity) for the catalyst, $[Mn(bpy)(CO)_3]^-$, and all adducts of the form Mn-CO_2-amine, Mn-amine-CO_2, Mn-MeOH-amine and Mn-amine-MeOH:

$[Mn(bpy)(CO)_3]^-$ (-1,1)

```
Mn 1.12200400 -0.00121200 -0.06343600
N -0.40852500 1.26712700 -0.12049400
C -1.68472900 -0.70968400 -0.08003200
C -1.68389400 0.71080900 -0.08003800
C -0.33160200 2.63492400 -0.10635500
C -2.84172100 1.53182000 -0.04366500
H -3.82482200 1.05962800 -0.01422100
C -1.42307900 3.46816700 -0.07300400
H -1.27287300 4.54941800 -0.07138400
C -2.73019900 2.90321700 -0.04292400
O 3.03079700 -2.06564900 -0.99424000
```

```
O 2.04039100 0.00819400 2.72642100
C 1.64966900 0.00406100 1.62382700
O 3.02931400 2.05751000 -1.00970200
C -0.33499600 -2.63560300 -0.10624900
H 0.67226000 -3.05341600 -0.12685900
C -1.42760700 -3.46731100 -0.07326500
H -1.27882600 -4.54876500 -0.07182500
C -2.84364400 -1.52913700 -0.04399600
H -3.82610400 -1.05558400 -0.01478200
C -2.73404000 -2.90068300 -0.04341500
H -3.62138900 -3.53618400 -0.01708900
C 2.27312900 1.24506400 -0.64091800
N -0.40989500 -1.26757300 -0.12022700
C 2.27387700 -1.25118300 -0.63153000
H 0.67628600 3.05123100 -0.12707700
H -3.61669700 3.53989700 -0.01624600
```

1.1 Mn-CO_2-DETA (-1,1)

```
Mn -1.37807000 -1.16169400 -0.21214300
N -2.51727500 0.48280300 0.18636500
C -0.79437000 1.55341100 -1.04482400
C -2.01551400 1.66759600 -0.26074600
C -3.67021200 0.49715300 0.88817800
C -2.67274100 2.88397200 0.00339200
H -2.25592800 3.81942900 -0.36812600
C -4.36174400 1.65949600 1.18854700
H -5.28613100 1.60496800 1.76536600
C -3.84885000 2.88731200 0.73650200
O 0.73276800 -3.20140800 -0.55621500
O -3.08096600 -2.25450300 -2.36883600
C -2.41040400 -1.78672800 -1.53984200
O -2.56116400 -2.72055900 2.00372400
C 0.77452900 0.08855100 -1.94725000
H 1.12892900 -0.93572300 -2.04775700
C 1.49074900 1.12395300 -2.52237900
H 2.41253900 0.88775800 -3.05372100
C -0.12029000 2.64897600 -1.61418000
H -0.50217300 3.65878300 -1.46717400
C 1.03359400 2.43997700 -2.35516200
H 1.57316600 3.28280800 -2.79153000
C -2.10520200 -2.11456900 1.12054700
N -0.34505000 0.27665900 -1.21469400
C -0.09765100 -2.39721400 -0.42407300
H -4.04040700 -0.47190200 1.22454800
H -4.36623400 3.82419600 0.95176400
C -0.01679500 -0.37466300 1.61578400
O -0.02428300 0.85471500 1.67372900
O 0.56515100 -1.23834300 2.28313500
N 3.49754200 -0.60078800 1.80324000
C 3.19497300 -0.86707600 0.39953500
H 2.69170600 -1.84604700 0.36304000
H 2.48850500 -0.14073500 -0.03571900
C 4.00607900 0.72751700 2.13386600
H 4.09413900 0.76870100 3.23404900
H 5.03760000 0.83804000 1.74818600
C 3.18532000 1.94692900 1.67920400
H 2.11599900 1.70519600 1.79561100
H 3.40267800 2.78657700 2.37574400
C 4.42571500 -0.92522600 -0.49611200
H 4.88379200 0.07552600 -0.54601000
H 5.17250400 -1.60205700 -0.02706300
H 2.61770700 -0.74785600 2.30531000
N 4.03799600 -1.31596000 -1.85445700
H 4.86384600 -1.37578600 -2.45064700
H 3.64646300 -2.25981700 -1.83602600
N 3.43124900 2.29535700 0.27814400
H 2.69982900 2.91551600 -0.06919900
H 4.30680500 2.81287100 0.19251300
```

1.2 Mn-DETA-CO_2 (-1,1)

```
Mn 1.91440900 -0.84017800 -0.34998300
N 1.78629800 1.12568600 -0.19081600
C 0.75664200 0.61944700 1.86914600
C 1.16780500 1.62935400 0.95432500
C 2.18540000 2.05568500 -1.12145100
C 0.96038000 3.01824000 1.13582800
H 0.45272600 3.36135900 2.03809300
C 2.00894800 3.41033700 -0.98371900
H 2.35785700 4.07603100 -1.77554400
C 1.37184800 3.92234300 0.18275700
O -0.23048000 -2.55957500 -1.36946600
O 3.72084900 -2.96854100 0.61566800
C 3.03571400 -2.10001700 0.23784200
O 3.27658000 -0.71281800 -2.97799200
C 0.62525000 -1.70165700 2.21628100
H 0.85373600 -2.69681400 1.83078800
C -0.03538300 -1.54107700 3.41279500
H -0.33643500 -2.41926600 3.98628300
C 0.09330700 0.84023400 3.10330500
H -0.11470000 1.86133100 3.42060100
C -0.30697500 -0.22595200 3.87751700
H -0.82413900 -0.06475000 4.82554400
C 2.74965200 -0.76014400 -1.93773000
N 1.03315900 -0.66167400 1.43579500
C 0.64465100 -1.86877800 -0.98202100
H 2.66664000 1.65705900 -2.01432400
H 1.21059300 4.99387700 0.31190900
C -3.86789600 -1.51808100 -0.80492800
O -3.67277800 -2.40318000 0.00863400
O -4.77245600 -1.10007400 -1.50202400
N -2.45909200 -0.62553700 -0.99508500
C -2.15975900 0.10252900 0.26937400
H -2.02321800 -0.67382500 1.03498400
H -1.21737100 0.64623800 0.15429000
C -2.43902500 0.16422400 -2.25420800
H -2.52156500 -0.57276400 -3.06447500
H -3.35417400 0.76970100 -2.28359100
C -1.19300200 1.02672200 -2.45447100
H -0.30631100 0.47586200 -2.09911700
H -1.06275300 1.15082500 -3.54860800
C -3.25993700 1.07168800 0.67590800
H -3.39417600 1.81017200 -0.12849300
H -4.21721000 0.52274000 0.78374600
H -1.74886800 -1.36358200 -1.07935800
N -2.84645800 1.78179700 1.88264000
H -3.58985500 2.41163400 2.18366300
H -2.71205700 1.11951100 2.64847800
N -1.27539700 2.28742300 -1.72692300
H -0.35769800 2.72894300 -1.67763800
H -1.89026900 2.93920700 -2.21478700
```

1.3 Mn-MeOH-DETA (-1,1)

```
Mn 0.81196400 -0.73797300 -0.99470700
N 0.66565000 1.22577100 -0.81385400
C 2.76332600 1.01955600 0.24133800
C 1.71081000 1.88705200 -0.16839600
C -0.36745900 2.01775000 -1.25896300
C 1.68351100 3.28623300 0.04599500
H 2.51891800 3.75698800 0.56641700
C -0.43366600 3.37544400 -1.07420600
H -1.29658300 3.92285000 -1.45863500
C 0.62108100 4.04516600 -0.39249600
O 0.19546200 -2.99058400 0.79919000
O 2.16049000 -2.35026200 -3.06031300
C 1.62797900 -1.68034000 -2.26330000
O -1.79643500 -1.04148900 -2.36080500
C 3.48383300 -1.21783900 0.28399100
H 3.25924000 -2.25375200 0.02410200
C 4.65685200 -0.88633400 0.92326200
H 5.37616700 -1.66786500 1.17309000
C 3.95333500 1.41334300 0.90521600
H 4.11065100 2.46679900 1.13919100
C 4.90214800 0.47432100 1.24678700
H 5.82150500 0.77002800 1.75609200
C -0.77017600 -0.90724600 -1.81825500
N 2.53315100 -0.30701900 -0.06433600
C 0.42573900 -2.07965100 0.09925200
C 1.40885900 -0.55696800 3.09329100
H 1.32161200 -1.63876300 2.88533400
H 2.43614400 -0.24452600 2.83119400
H 1.27666400 -0.40830800 4.17720000
H -1.16644200 1.49998300 -1.78852900
O 0.42490800 0.20228200 2.42663400
H 0.45996300 -0.02180800 1.47657900
H 0.59110000 5.12379400 -0.22903600
N -2.57867300 0.37361400 0.84396800
C -3.64676400 0.74448100 -0.07353600
H -3.27574800 1.59432500 -0.67426800
H -3.92136000 -0.05396800 -0.78757500
C -2.75729500 -0.80688400 1.68228600
H -1.80708600 -0.93193400 2.22288300
```

```
H -3.52462500 -0.60191300 2.45336200
C -3.10658000 -2.13839800 1.00088100
H -2.51487400 -2.22923100 0.07654100
H -2.76298700 -2.95940100 1.66634800
C -4.91425200 1.19220500 0.64758300
H -5.34381900 0.32852700 1.17901100
H -4.62971500 1.93917500 1.41938700
H -1.70598100 0.26768900 0.32836900
N -5.90749700 1.66750100 -0.31619400
H -6.77206700 1.90614400 0.17079300
H -5.58492900 2.54524600 -0.72919800
N -4.52701600 -2.23393900 0.65562000
H -4.70761200 -3.11723200 0.17646300
H -5.07963500 -2.27593500 1.51408100
```

1.4 Mn-DETA-MeOH (-1,1)

```
Mn 1.99241400 -0.31424400 -0.01239600
N 0.63486600 -1.07734200 -1.24618100
C 0.00914700 1.17536500 -1.50629400
C -0.21134100 -0.17589700 -1.88414400
C 0.46960400 -2.40245600 -1.55521200
C -1.21575700 -0.61891200 -2.78230300
H -1.88372500 0.11772200 -3.22608500
C -0.48605200 -2.87711700 -2.41973800
H -0.55731500 -3.95100000 -2.60265000
C -1.36819400 -1.95836200 -3.05670400
O 2.89726800 0.92100000 2.51709800
O 4.54990300 -0.16408400 -1.45231800
C 3.51773100 -0.22086600 -0.90455800
O 2.45875700 -2.93264100 1.28833300
C 1.26604900 2.60260200 -0.12131900
H 2.06919000 2.70124200 0.60980000
C 0.56959300 3.70926700 -0.54177000
H 0.82369200 4.68971000 -0.13462000
C -0.73166100 2.28911300 -1.97660700
H -1.52560400 2.12339700 -2.70287600
C -0.46902200 3.55435000 -1.50362600
H -1.03978300 4.41566600 -1.85604500
C 2.28051900 -1.90216300 0.76510300
N 1.01552800 1.32892500 -0.55898600
C 2.55451000 0.43876600 1.50578600
C -4.04271000 2.04119100 -0.50636000
H -4.50610700 1.89350900 0.49080600
H -3.36174800 2.91387300 -0.42941500
H -4.85239800 2.32060400 -1.20267500
H 1.15049800 -3.09283400 -1.05654900
O -3.39933100 0.89319200 -0.98866600
H -2.71615800 0.62162400 -0.31366900
H -2.14485500 -2.30835100 -3.73959500
N -1.66915200 0.12176600 1.05890200
C -1.91638600 -1.29932500 1.31270600
H -1.38711800 -1.86371200 0.52738300
H -1.49594400 -1.62610500 2.28007800
C -1.85069500 1.02417400 2.20106300
H -2.22685100 1.99760500 1.84011600
H -2.64031200 0.61778400 2.85549400
C -0.58271600 1.29487100 3.01197500
H 0.17827400 1.69859500 2.32587800
H -0.80872300 2.10751800 3.73571800
C -3.40038000 -1.65645800 1.25308600
H -3.94224400 -1.10395000 2.04110600
H -3.80649700 -1.29528900 0.28818400
H -0.71016000 0.22246100 0.71854600
N -3.58122700 -3.08836000 1.49409400
H -4.57615800 -3.31558800 1.47803800
H -3.17886200 -3.61066300 0.71255100
N -0.03379600 0.09071100 3.62660000
H 0.92512000 0.26283900 3.92678800
H -0.56628800 -0.15905300 4.46050600
```

2.1 Mn-CO_2-DEA (-1,1)

```
Mn -1.82839100 -0.91786900 0.01542800
N -2.21463900 1.08293300 0.31149100
C -0.50855900 1.30507500 -1.32839100
C -1.40961800 1.94082600 -0.37138900
C -3.11294200 1.59368700 1.17744300
C -1.49649900 3.33122600 -0.18117700
H -0.84557900 4.00091500 -0.74187600
C -3.24553900 2.95297400 1.41750100
H -3.98784400 3.30564200 2.13485400
C -2.41368000 3.84508800 0.72339200
O -0.55760800 -3.56993500 -0.28977300
O -4.19866000 -1.53412600 -1.65038100
C -3.26133600 -1.26124800 -1.01883700
O -3.00823900 -1.70098400 2.61152100
C 0.13854600 -0.70772800 -2.30643800
H 0.02081400 -1.79096900 -2.33811900
C 1.02857200 -0.07062400 -3.15669400
H 1.61283000 -0.65897000 -3.86547000
C 0.37863400 2.01205600 -2.15831700
H 0.44990500 3.09620900 -2.08200600
C 1.15940600 1.32522700 -3.07672500
H 1.85549500 1.86128000 -3.72425500
C -2.55998500 -1.40255400 1.58127200
N -0.61222100 -0.05132900 -1.39651300
C -1.07540500 -2.53672000 -0.17433100
H -3.74175500 0.87257500 1.70083200
H -2.48894600 4.92205300 0.88499900
C 0.05702900 -0.49029400 1.40853700
O 0.51106900 0.64497000 1.27165300
O 0.46726100 -1.44550100 2.10627600
N 3.88563900 0.13178800 -0.98634500
C 3.75402600 0.98250400 0.19560200
H 3.67881000 2.02644400 -0.15812500
H 2.84818600 0.77991000 0.79462400
C 3.94775500 -1.31410100 -0.78409700
H 3.94693700 -1.77681000 -1.78635300
H 4.91685100 -1.58314400 -0.32573800
C 2.83882300 -1.97054100 0.03980200
H 1.86322000 -1.57499000 -0.29660700
H 2.83246400 -3.05850700 -0.19427500
C 4.96653400 0.89635900 1.10722300
H 5.00092200 -0.10539600 1.56962700
H 5.88721400 1.02795300 0.50216600
H 3.10703700 0.33501000 -1.60960500
O 4.83699000 1.90898800 2.09519400
H 5.52956100 1.77251300 2.75392500
O 3.02142900 -1.75422700 1.42021100
H 2.11968300 -1.62949500 1.81140100
```

2.2 Mn-DEA-CO_2 (-1,1)

```
Mn -1.00182700 -0.67279800 1.04381700
N -1.81923200 1.12377700 0.74135100
C -2.84094400 0.03699800 -1.07448100
C -2.69211200 1.24063100 -0.32299300
C -1.62074100 2.22934500 1.51655100
C -3.34531900 2.46698000 -0.60143500
H -4.02854700 2.52679000 -1.44927000
C -2.22599600 3.44321900 1.28506900
H -2.01256100 4.28553900 1.94531700
C -3.11956700 3.57227000 0.18993400
O 0.57312600 -3.15277700 0.73884700
O -2.35058400 -1.48666000 3.53079800
C -1.86020600 -1.15905800 2.52230900
O 1.40371700 0.51593500 2.27312200
C -2.16044800 -2.20098700 -1.27688100
H -1.55400600 -3.01847700 -0.88678000
C -2.94361800 -2.39016400 -2.38971000
H -2.95063600 -3.36636300 -2.87817100
C -3.66766900 -0.11127900 -2.21375300
H -4.25565800 0.74072400 -2.55723500
C -3.72957400 -1.31423700 -2.88278900
H -4.36284300 -1.43742100 -3.76318700
C 0.44106900 0.04511600 1.79324500
N -2.07433800 -1.01561600 -0.59298700
C -0.06739200 -2.17397100 0.86277500
H -0.93367700 2.10216100 2.35390600
H -3.61647300 4.52160600 -0.01888400
N 3.03462600 0.61455500 -0.48354100
C 2.88536600 -0.79318500 -0.82280300
H 1.83156300 -1.10047900 -0.95418200
H 3.39250600 -0.97926200 -1.78573200
C 2.48336900 1.57932400 -1.42713400
H 2.92404700 2.56915900 -1.20737000
H 2.81955600 1.30032900 -2.44115900
C 0.96221800 1.74997800 -1.43699700
H 0.61753800 2.01743200 -0.42155800
H 0.71924200 2.60736300 -2.09310200
C 3.51190300 -1.68786500 0.24506600
H 4.59485800 -1.49565900 0.29974700
H 3.08411300 -1.42565500 1.23304000
```

H 2.66500300 0.79074200 0.45104500
O 3.33483300 -3.05919000 -0.04628500
H 2.39550000 -3.25596500 0.10326000
O 0.26264500 0.61749300 -1.91441800
H -0.05618400 0.10568400 -1.14363600
C 5.57775300 1.32736000 -0.40811300
O 5.34359600 2.32288600 0.15187600
O 5.96471400 0.38104100 -0.96924000

2.3 Mn-MeOH-DEA (-1,1)

Mn 0.79237600 -0.75009400 -0.98661700
N 0.65158400 1.21647900 -0.83217500
C 2.75312400 1.01933500 0.21743000
C 1.70003300 1.88358600 -0.19853200
C -0.38149600 2.00505000 -1.28365800
C 1.67540200 3.28497300 0.00038400
H 2.51318000 3.76031400 0.51272400
C -0.44533500 3.36481500 -1.11351700
H -1.30868100 3.90910500 -1.50133800
C 0.61233300 4.04046300 -0.44267600
O 0.16493700 -2.96971200 0.84395300
O 2.12680100 -2.39751200 -3.03400700
C 1.60029100 -1.71385800 -2.24494400
O -1.82557600 -1.06298000 -2.33151400
C 3.47013700 -1.21849400 0.28475000
H 3.24259700 -2.25717300 0.03890200
C 4.64673900 -0.88084300 0.91420400
H 5.36601500 -1.66033900 1.17037100
C 3.94658600 1.41947900 0.87101200
H 4.10659900 2.47546200 1.09126600
C 4.89557500 0.48316700 1.21971100
H 5.81769100 0.78368200 1.72112600
C -0.79421800 -0.92521700 -1.79929400
N 2.51941600 -0.31039100 -0.07051300
C 0.40312600 -2.07210400 0.12902100
C 1.42631300 -0.52470800 3.10302100
H 1.34664600 -1.60963500 2.90854800
H 2.45161700 -0.20821200 2.83821400
H 1.29130700 -0.36327000 4.18469600
H -1.18295500 1.48280500 -1.80504600
O 0.43813800 0.21914300 2.42498100
H 0.47922400 -0.01523300 1.47757500
H 0.58422000 5.12081400 -0.29079700
N -2.55993700 0.40264200 0.86742600
C -3.62986800 0.74125800 -0.05881500
H -3.28943100 1.59711000 -0.66532800
H -3.88592600 -0.07070400 -0.76621900
C -2.72144300 -0.77765500 1.70928000
H -1.76218200 -0.91263400 2.23056200
H -3.47990300 -0.58510700 2.49051600
C -3.08864800 -2.09335600 1.02822600
H -2.47199300 -2.22446200 0.12206600
H -2.84086100 -2.92863700 1.71442000
C -4.90299900 1.16509400 0.65599700
H -5.31966000 0.30437100 1.20836500
H -4.65399900 1.95494100 1.39333200
H -1.68105400 0.31718300 0.35882700
O -5.81609100 1.62922100 -0.32708000
H -6.65837000 1.80973400 0.10918700
O -4.47529800 -2.09847000 0.70989400
H -4.64812600 -2.85229400 0.13089500

2.4 Mn-DEA-MeOH (-1,1)

Mn -0.42017100 -1.45283300 0.11851400
N 1.19706100 -1.11081800 -1.00661600
C 2.07353100 -0.28205600 1.01046700
C 2.29855500 -0.57790200 -0.36776000
C 1.31926900 -1.40910800 -2.33208900
C 3.51319800 -0.36283400 -1.06475600
H 4.37075700 0.04417600 -0.52871600
C 2.47084100 -1.20354100 -3.05753300
H 2.49247800 -1.46124000 -4.11771800
C 3.61056000 -0.66652100 -2.40574500
O -2.79587300 -1.19751700 1.85499800
O -0.38732800 -4.39764400 0.11551000
C -0.35553300 -3.23010100 0.14153400
O -2.25091500 -1.08722200 -2.16697800
C 0.50163600 -0.31978600 2.75276800
H -0.50879800 -0.56760300 3.07769400
C 1.39583400 0.23676600 3.63530500
H 1.08168100 0.42798500 4.66315300
C 3.02806500 0.29108800 1.88231700
H 4.01995200 0.53064800 1.49838500
C 2.70785100 0.55396000 3.19576700
H 3.43594800 1.00117400 3.87472200
C -1.51628200 -1.23722500 -1.26340700
N 0.79263700 -0.59741200 1.44168000
C -1.84272500 -1.30869300 1.17494100
H 0.43180100 -1.83132500 -2.80504100
H 4.54166300 -0.50084100 -2.95094600
N -2.81051500 2.00357400 -1.54234600
C -3.15168000 1.81734000 -0.13984000
H -2.37006000 1.29860600 0.44763500
H -3.28804100 2.80765500 0.33000200
C -1.63932700 2.81751800 -1.82759000
H -1.66561400 3.12369900 -2.89060500
H -1.70438000 3.74578400 -1.23299400
C -0.27176200 2.17580600 -1.58488900
H -0.18817500 1.23815800 -2.16145600
H 0.51290900 2.85811200 -1.95695400
C -4.44901100 1.02443900 -0.00082500
H -5.27165000 1.57884000 -0.48261700
H -4.34486500 0.06556200 -0.54806600
H -2.71731000 1.09424300 -1.99486100
O -4.80272800 0.81096600 1.35005000
H -4.15945900 0.17721800 1.70804900
O -0.00920500 1.91790700 -0.21233400
H -0.19613600 0.97147400 -0.01543900
C 3.00096600 3.33058200 -0.18203600
H 3.92151800 3.81806100 0.18170400
H 3.27795000 2.31515000 -0.52298600
H 2.66641900 3.89524100 -1.07816600
O 2.05239900 3.32220700 0.85188700
H 1.29665900 2.77467200 0.54849900

3.1 Mn-CO_2-PMDETA (-1,1)

N -3.18026000 1.19968100 1.38533200
C -3.56670100 1.26260900 -0.01939100
H -3.56518200 2.32915500 -0.30151200
H -2.82943500 0.76776900 -0.68292100
C -3.14610600 -0.13725000 1.97826100
H -2.77004200 -0.01402900 3.00662300
H -4.17846400 -0.50920100 2.08231600
C -2.28641900 -1.20894300 1.27830100
H -1.43533800 -0.71666600 0.78596900
H -1.85584200 -1.88489000 2.05113900
C -4.94640300 0.66921600 -0.29067900
H -4.89568400 -0.40794700 -0.08748600
H -5.68500600 1.11054100 0.41887400
N -5.36112200 0.83306300 -1.67895000
N -3.00532800 -1.99037600 0.27156000
C -5.80782900 2.17777500 -1.98788000
H -6.02124800 2.27048300 -3.06472000
H -6.73355800 2.46245100 -1.43413800
H -5.03209400 2.91591100 -1.73744800
C -6.34457100 -0.15549200 -2.07671100
H -6.58001500 -0.04812100 -3.14790700
H -5.95034700 -1.17064100 -1.91363700
H -7.30465700 -0.07055400 -1.51506100
C -1.95997300 1.95361000 1.63345000
H -2.09871500 2.99929600 1.31058900
H -1.74098500 1.96462100 2.71370600
H -1.07562000 1.55620600 1.10103000
C -2.09844600 -2.65012800 -0.65086400
H -1.42705500 -3.38235300 -0.14249600
H -2.67460100 -3.20760300 -1.40739500
H -1.46413900 -1.92017500 -1.17314100
C -3.92220900 -2.94332600 0.86565600
H -4.49347200 -3.46273700 0.08010100
H -3.39849500 -3.71758800 1.47554700
H -4.64538500 -2.43873100 1.52328000
Mn 2.83905400 -0.41380700 -0.76212800
N 2.55489700 1.43973100 0.03423700
C 1.50874500 0.17319300 1.74454200
C 1.84934800 1.47731800 1.19946200
C 2.94064300 2.60974800 -0.51906200
C 1.52212900 2.69941000 1.81696400
H 0.96067300 2.70377000 2.75014700
C 2.64129200 3.84505900 0.03121100
H 2.97550600 4.75479900 -0.46967000
C 1.90986000 3.89320300 1.23096800

O 2.55927000 -3.15396800 -1.83409800
O 5.68543300 -0.71342600 -0.02122000
C 4.55608300 -0.58515300 -0.27377700
O 3.29545500 0.65115200 -3.48266700
C 1.68857300 -2.13216800 1.45830200
H 2.03949600 -2.95467900 0.83495500
C 1.02908000 -2.38459200 2.64799700
H 0.85963100 -3.41578800 2.96112300
C 0.83206100 -0.01734300 2.96363400
H 0.50573500 0.84497900 3.54296800
C 0.58464300 -1.29965400 3.42388500
H 0.05671600 -1.46096600 4.36528700
C 3.12995300 0.22947100 -2.41016800
N 1.93188000 -0.88445500 0.99338200
C 2.68306600 -2.07776600 -1.41219000
H 3.50641900 2.53893300 -1.44852400
H 1.65627600 4.84809200 1.69519700
C 0.55429800 -0.20114700 -1.51274600
O -0.09414300 0.49603200 -0.73421100
O 0.25791200 -0.81836000 -2.53699200

3.2 Mn-PMDETA-CO_2 (-1,1)

Mn 3.03363300 -0.17984300 -0.35129900
N 2.15386000 1.49317800 0.23428700
C 1.13796400 0.08201700 1.82415500
C 1.29474500 1.40438500 1.33289500
C 2.31951100 2.74887800 -0.30296100
C 0.66130800 2.55155800 1.87290800
H 0.00523700 2.43335700 2.73620400
C 1.71442800 3.88180600 0.17863900
H 1.90225000 4.83777800 -0.31434200
C 0.85627500 3.79303600 1.31403000
O 2.52565300 -2.60581500 -1.95303200
O 5.59564500 -1.07264000 0.78710200
C 4.58086800 -0.68615200 0.35107500
O 4.26768900 1.10009400 -2.72057100
C 1.80385200 -2.15580400 1.54266300
H 2.41643700 -2.86474900 0.98300400
C 0.99953900 -2.58431000 2.57305700
H 0.97310500 -3.64363100 2.83367700
C 0.29339500 -0.30886900 2.89695500
H -0.29921900 0.44935300 3.41034300
C 0.21413300 -1.62958300 3.27678900
H -0.44054600 -1.93800800 4.09414200
C 3.78564900 0.60137200 -1.78010700
N 1.89234600 -0.85686600 1.13885500
C 2.72619300 -1.63629400 -1.32635000
H 2.98353000 2.80947000 -1.16560100
H 0.36766100 4.67955100 1.72215000
N -0.81975900 -0.66089800 -1.77596000
C -1.90004700 0.31568600 -1.71609000
H -1.79655100 0.96065900 -2.60315100
H -2.89900200 -0.15488100 -1.77751600
C -0.84981600 -1.71197300 -0.75920700
H 0.03071400 -2.34501300 -0.93373300
H -0.68967800 -1.26214600 0.22906000
C -5.16056700 0.46217600 -0.13315800
O -5.14859400 0.42458800 1.03219800
O -5.32229000 0.43268500 -1.28740300
C -2.09162300 -2.61678400 -0.72419500
H -2.41978100 -2.81526400 -1.75707500
H -1.79620000 -3.60730000 -0.30401900
C -1.82664600 1.17674700 -0.45699500
H -1.74170800 0.52655900 0.42433700
H -0.89484500 1.78085100 -0.48786600
N -3.00312300 2.01889400 -0.25548900
N -3.23203200 -2.07646900 0.01454000
C -3.22044900 2.97255100 -1.32868200
H -4.10402000 3.58895500 -1.10305400
H -2.35483500 3.65698500 -1.47662100
H -3.41277700 2.45387900 -2.27825200
C -2.96761600 2.67221100 1.04167400
H -3.90805000 3.21840400 1.21365100
H -2.86094800 1.91776300 1.83534300
H -2.12526600 3.38904800 1.13851100
C -0.61492500 -1.17507500 -3.11659100
H -0.40953700 -0.34289700 -3.80913600
H 0.25423200 -1.84936700 -3.12677400
H -1.48712900 -1.73263500 -3.52760600
C -4.45407200 -2.78652800 -0.31576300
H -4.40001400 -3.87365700 -0.07565500
H -5.30050700 -2.36714200 0.24947200
H -4.67503100 -2.68719800 -1.38971100
C -3.00433500 -2.06926800 1.44852800
H -3.85203800 -1.59138300 1.95958700
H -2.87797000 -3.09637400 1.86391100
H -2.09879500 -1.50176600 1.69959000

3.3 Mn-MeOH-PMDETA (-1,1)

Mn 1.91042800 -0.59728500 -1.13343200
N 2.00735700 1.35498100 -0.81245200
C 3.78451500 0.76073700 0.61570600
C 2.98066200 1.80543100 0.07772900
C 1.21183400 2.32108400 -1.38036400
C 3.11461600 3.17813900 0.39849700
H 3.88546200 3.48233700 1.10813000
C 1.30597400 3.66087500 -1.09876000
H 0.62633100 4.35792600 -1.59285200
C 2.28522100 4.11761600 -0.17264100
O 0.85961100 -3.02190500 0.17405100
O 3.40095500 -2.01498500 -3.24067300
C 2.82540400 -1.41391400 -2.41837800
O -0.56681700 -0.39770300 -2.73938800
C 4.15023700 -1.56075600 0.61531900
H 3.84626400 -2.53326900 0.22466800
C 5.18807100 -1.45374100 1.51279200
H 5.71547300 -2.35179100 1.83863900
C 4.84581800 0.92560200 1.54209300
H 5.09882000 1.92764500 1.89037500
C 5.55012800 -0.16808200 1.99591900
H 6.36803700 -0.04910900 2.70932700
C 0.41929400 -0.46984700 -2.11361300
N 3.43484700 -0.49496500 0.15830000
C 1.25965100 -2.04960400 -0.34189500
C 1.44646800 -0.60595400 2.88304800
H 1.21278500 -1.64642700 2.59313400
H 2.54697900 -0.49664100 2.88474000
H 1.08830100 -0.45311800 3.91396400
H 0.46842000 1.96418100 -2.09195000
O 0.80995500 0.33961300 2.05420200
H 1.00636700 0.10868200 1.12304400
H 2.37923300 5.17733700 0.07119400
N -2.88465100 1.26214000 0.92251900
C -3.97355700 1.09344700 -0.02958500
H -4.11798500 2.06993200 -0.52187300
H -3.73324500 0.36701300 -0.83348700
C -2.52767800 0.09389400 1.72923200
H -1.71554000 0.40824700 2.40069000
H -3.38248300 -0.17649400 2.37004400
C -2.02474300 -1.14704600 0.97794800
H -1.32351600 -0.82360200 0.19642800
H -1.41849600 -1.76785900 1.67468600
C -5.28428900 0.65894000 0.61998700
H -5.14560100 -0.34223700 1.04708600
H -5.51848200 1.34466000 1.46770800
N -6.38332600 0.57517700 -0.33366400
N -3.07976000 -1.94208800 0.35255500
C -6.89775100 1.87260400 -0.73002200
H -7.66835700 1.75340000 -1.50800500
H -7.35769700 2.43133300 0.11873500
H -6.09861100 2.50201600 -1.14734300
C -7.44719400 -0.29237700 0.13461400
H -8.22464900 -0.39198700 -0.63983000
H -7.05167300 -1.29670000 0.35262900
H -7.94448400 0.08349900 1.05945800
C -1.73309500 1.88750000 0.29777200
H -2.00629300 2.88337300 -0.08935000
H -0.91892500 2.00667000 1.02635000
H -1.32242700 1.31015400 -0.55642200
C -2.59939300 -2.71093100 -0.78070200
H -1.83680200 -3.47150700 -0.49910700
H -3.43958100 -3.23875800 -1.26072400
H -2.14200700 -2.04164900 -1.52394200
C -3.77916600 -2.78038800 1.30412600
H -4.63537200 -3.27707700 0.82020400
H -3.12660100 -3.57328400 1.74094900
H -4.17141900 -2.18103000 2.13922400

3.4 Mn-PMDETA-MeOH (-1,1)

Mn -2.36673100 -0.90781500 -0.17712500
N -1.22262400 -0.86636800 1.43820400

C -1.73610100 1.43285200 1.41621400
C -1.02911000 0.37466800 2.04833900
C -0.54920100 -1.92619700 1.99788200
C -0.19965000 0.51966900 3.18855300
H -0.07775100 1.51018200 3.62933200
C 0.25898700 -1.83137700 3.10190900
H 0.75379300 -2.72901600 3.47787700
C 0.44763400 -0.56672000 3.73081600
O -1.53161500 -0.39899100 -2.95265200
O -5.25521800 -1.09481500 -0.75111700
C -4.11404400 -1.03245800 -0.49654400
O -1.97125800 -3.81361800 -0.58779400
C -3.21096400 1.96700200 -0.33665300
H -3.78565600 1.60214000 -1.19000200
C -3.23755500 3.29723200 0.01405700
H -3.84247200 3.99729600 -0.56459400
C -1.71972300 2.79685900 1.81149400
H -1.11142200 3.09567700 2.66618600
C -2.46229500 3.73133000 1.12427700
H -2.45313100 4.78160100 1.42279100
C -2.13846100 -2.67019400 -0.41250300
N -2.48617500 1.02324700 0.32674000
C -1.84147800 -0.59160000 -1.83792100
C 6.40193900 -0.00029400 -0.07665000
H 6.14048700 0.05279700 -1.15379800
H 6.63319400 1.03429900 0.25237800
H 7.33716200 -0.58105700 0.00142300
H -0.68613500 -2.88684900 1.50423600
O 5.41159700 -0.61852000 0.70169900
H 4.58394400 -0.06837900 0.62371400
H 1.08890200 -0.46281200 4.60794900
N 3.11231200 0.86638000 0.40528300
C 2.09233100 -0.18010500 0.25321800
H 2.07708100 -0.74592000 1.19582800
H 1.08531700 0.24846500 0.12640800
C 3.34453400 1.72393700 -0.76464100
H 4.21761900 2.35467300 -0.52969800
H 3.65229500 1.08758500 -1.60741900
C 2.20032500 2.64995600 -1.19530300
H 1.79762500 3.15338900 -0.30307600
H 2.62920500 3.46047800 -1.83091300
C 2.38043300 -1.14018600 -0.90104800
H 2.14832900 -0.64266800 -1.85124100
H 3.46675200 -1.37835600 -0.91005900
N 1.56277200 -2.34157100 -0.81261900
N 1.09817600 1.97817700 -1.86946500
C 2.10141100 -3.33302400 0.09229900
H 1.37534000 -4.14813000 0.23915100
H 3.05247000 -3.78427000 -0.27737800
H 2.30177600 -2.88767200 1.07717600
C 1.20068600 -2.90288500 -2.09649700
H 0.49240900 -3.73307300 -1.95007500
H 0.69613100 -2.14293400 -2.71141700
H 2.07328900 -3.29434900 -2.67087700
C 2.91881200 1.62155800 1.63635600
H 2.94485900 0.93509600 2.49539000
H 3.72598300 2.36137100 1.75168700
H 1.94810800 2.15369900 1.67757500
C -0.14071400 2.72767300 -1.77969600
H -0.08611800 3.71579500 -2.29403800
H -0.95808000 2.15311300 -2.23545400
H -0.40233400 2.90578000 -0.72775000
C 1.40209200 1.62917600 -3.24373900
H 0.58770400 1.01729900 -3.65852500
H 1.53372400 2.52650100 -3.89308100
H 2.32928900 1.04091400 -3.30496600

4.1 Mn-CO_2-NEt_3 (-1,1)

Mn 1.30639100 -0.99217800 0.69995500
N 2.65009700 0.07512100 -0.37446500
C 1.25001300 1.87051200 0.28801900
C 2.38470000 1.41002500 -0.49255300
C 3.72532000 -0.41961000 -1.03141500
C 3.18843800 2.24973100 -1.28706700
H 2.95336100 3.31097900 -1.36346400
C 4.55024800 0.35220100 -1.83001400
H 5.39720300 -0.11244200 -2.33719100
C 4.27403100 1.72518000 -1.96839900
O -1.25461300 -2.21456500 1.53015800
O 2.68524800 -1.30752800 3.29513300
C 2.16292000 -1.14877300 2.26663700
O 2.22071000 -3.57894200 -0.40220600
C -0.39935000 1.24801200 1.81293400
H -0.86594600 0.44098600 2.37785700
C -0.86111400 2.54807700 1.92736000
H -1.69860200 2.76700100 2.59112900
C 0.82779300 3.21224500 0.35837200
H 1.34147300 3.97465800 -0.22637600
C -0.23694600 3.55825800 1.17404800
H -0.57691200 4.59373200 1.23494500
C 1.86781300 -2.56411200 0.04207300
N 0.62487400 0.89434100 1.00651400
C -0.24021200 -1.73645300 1.21544200
H 3.91435300 -1.48627000 -0.90869800
H 4.90303800 2.36431700 -2.59077500
C 0.09286100 -0.86600500 -1.39598500
O 0.08399900 0.28322000 -1.82574000
O -0.37471800 -1.93348000 -1.79361300
N -3.72958600 -0.11804100 -0.52958500
C -4.02139700 1.00436700 -1.41386300
H -4.05263900 0.61752600 -2.44298200
H -5.03308500 1.43161700 -1.22628900
C -3.81013100 0.19091900 0.89540600
H -3.37290900 -0.66047900 1.44197100
H -3.14865700 1.04584000 1.09729000
C -4.40718300 -1.35692200 -0.89471500
H -4.24385000 -2.06806200 -0.06801400
H -5.51144800 -1.22680100 -0.97123500
C -3.88458300 -1.98779400 -2.18525900
H -4.35430100 -2.97288700 -2.33961500
H -4.12065000 -1.37688500 -3.07105800
H -2.79178400 -2.11407900 -2.13948100
C -5.20581500 0.49603700 1.45738800
H -5.89242300 -0.35659700 1.33340000
H -5.13936900 0.71606400 2.53515700
H -5.66386500 1.37094000 0.96899900
C -2.97282700 2.11352800 -1.35555700
H -3.20627100 2.89496900 -2.09673700
H -2.93242000 2.60012600 -0.36899000
H -1.97423200 1.70407600 -1.57292100

4.2 Mn- NEt_3-CO_2 (-1,1)

Mn -1.84550000 0.81383900 -0.52872800
N -1.74231100 -1.15818300 -0.27865300
C -1.07610700 -0.56297400 1.89565700
C -1.37217500 -1.60893000 0.98287500
C -2.02617000 -2.10690900 -1.22084500
C -1.29046600 -2.99783100 1.26668600
H -0.99809800 -3.31782500 2.26761200
C -1.94984100 -3.46070000 -0.99652100
H -2.18239000 -4.15446000 -1.80641400
C -1.56951500 -3.92932600 0.29332700
O -1.01744000 3.64432200 -0.78533500
O -4.73923800 1.29914400 -0.67709400
C -3.59053700 1.09442300 -0.58822900
O -1.36385000 0.55974800 -3.43515800
C -0.92537700 1.76692000 2.17413600
H -1.05080700 2.75747500 1.73606200
C -0.48898500 1.63738000 3.46967400
H -0.26539300 2.53333900 4.05189700
C -0.64052300 -0.74613600 3.23385600
H -0.54277200 -1.76046900 3.62340700
C -0.33799600 0.33523200 4.02874000
H 0.00379500 0.20116500 5.05687100
C -1.56849600 0.66130700 -2.28697000
N -1.22012300 0.71150700 1.35042300
C -1.35767400 2.53117300 -0.67449300
H -2.31961800 -1.72554100 -2.19980200
H -1.50338000 -4.99859900 0.50413400
C 5.08097700 0.35610600 0.13910900
O 4.82273200 0.32077000 1.27750100
O 5.53120900 0.42987200 -0.93560700
N 2.68506800 -0.09325100 -0.70516000
C 1.86980100 -0.60216200 0.40258800
H 1.93957900 0.12186800 1.22589400
H 0.80281400 -0.63555200 0.12012300
C 2.85809500 -1.04067900 -1.80911000
H 3.52614300 -0.56038400 -2.54223300
H 3.40960500 -1.91536900 -1.43001800
C 2.23363100 1.21786500 -1.18371200
H 2.91673200 1.51248900 -1.99775700
H 1.21713900 1.15618200 -1.62211900

```
C 2.21810100 2.31320800 -0.12244600
H 1.95892700 3.27297000 -0.59413100
H 1.46031600 2.12439000 0.65088400
H 3.19597600 2.42268600 0.37224700
C 1.58039200 -1.51711900 -2.51165200
H 1.01316700 -0.68560700 -2.95437400
H 1.83997200 -2.21658900 -3.32260900
H 0.90309600 -2.04303500 -1.82116300
C 2.29992800 -1.96440200 0.93030500
H 1.73165100 -2.19046700 1.84484200
H 2.10072100 -2.77750900 0.21603300
H 3.37219000 -1.97715200 1.18023000
```

4.3 Mn-MeOH-NEt_3 (-1,1)

```
Mn 0.98143700 -0.90558100 -0.94118700
N 1.05029000 1.07044400 -1.01800600
C 2.73554300 0.80771800 0.60840800
C 1.97352400 1.70793100 -0.18858300
C 0.28255100 1.89014800 -1.81153300
C 2.10028100 3.11805400 -0.16465400
H 2.83340600 3.57133700 0.50428300
C 0.37524200 3.25899600 -1.82254500
H -0.27735900 3.83093700 -2.48502200
C 1.31251700 3.90888900 -0.97104200
O -0.29043800 -2.92135500 0.79143600
O 2.63784400 -2.80342800 -2.46831500
C 1.99271100 -2.02033800 -1.88598600
O -1.26975700 -1.07599900 -2.85396900
C 3.08063600 -1.45882500 1.12788800
H 2.79339000 -2.49368200 0.93397500
C 4.05565000 -1.15661800 2.05099000
H 4.55087100 -1.96092300 2.59743500
C 3.73353000 1.17270600 1.54834400
H 3.96983000 2.22745000 1.69318200
C 4.39583300 0.20482300 2.27113500
H 5.16417400 0.47810300 2.99713600
C -0.37755700 -1.00561700 -2.10201700
N 2.41025000 -0.51909100 0.40460000
C 0.20042200 -2.10892900 0.10485400
C 0.23129800 -0.05043500 2.95089200
H 0.02230600 -1.13415500 2.88983900
H 1.32865000 0.07861000 2.99401500
H -0.19120300 0.32339300 3.89749900
H -0.43837400 1.38691700 -2.45495600
O -0.35842600 0.67251600 1.89413500
H -0.06353700 0.27438700 1.05069100
H 1.40335700 4.99643700 -0.95905200
N -3.82279900 0.27221900 0.66560700
C -5.24619100 0.37090000 0.37824900
H -5.47182200 1.43175400 0.18332600
H -5.52186000 -0.16720700 -0.55876200
C -3.32997800 -1.07323800 0.95071900
H -2.26835600 -0.97104000 1.21660200
H -3.83544500 -1.44789900 1.85707200
C -3.00254900 1.01768200 -0.28671100
H -1.99000100 0.59039400 -0.27581500
H -3.37141400 0.89700800 -1.32934300
C -2.90945900 2.50176300 0.05981000
H -2.30820800 3.03958300 -0.68941500
H -3.90257000 2.97782600 0.10212700
H -2.42624800 2.62298400 1.04143300
C -3.47972800 -2.10935500 -0.17000000
H -2.99362700 -1.76941700 -1.09662000
H -2.99931900 -3.05432900 0.12715300
H -4.53713800 -2.32256500 -0.39592600
C -6.14055900 -0.11139800 1.51629800
H -7.19644800 0.10659400 1.29170400
H -6.05625300 -1.19677900 1.68087800
H -5.87299300 0.39577600 2.45744300
```

4.4 Mn-NEt_3-MeOH (-1,1)

```
Mn 2.27907800 -0.46981300 -0.11059800
N 1.57364400 1.38245700 0.15953900
C 0.15461400 0.31134600 1.69021200
C 0.52922700 1.52615900 1.05558100
C 1.99448800 2.49399800 -0.50769700
C -0.07034900 2.79457400 1.27359400
H -0.90493800 2.87359100 1.97069200
C 1.44996700 3.74483000 -0.32802600
H 1.84083700 4.59111400 -0.89547600
C 0.38023800 3.90366200 0.59500400
O 3.19443900 -3.28599400 -0.10846700
O 5.05930800 0.49246000 -0.35845000
C 3.96201600 0.10403100 -0.23565400
O 1.70188800 -0.51773900 -2.99349200
C 0.58511100 -1.99572300 1.85761100
H 1.17065200 -2.85290800 1.52523400
C -0.40340600 -2.16125700 2.79372200
H -0.59193100 -3.15712200 3.19960700
C -0.87890200 0.18521400 2.65166200
H -1.45134500 1.07071300 2.92772900
C -1.16919100 -1.03548900 3.21622200
H -1.96341700 -1.14069300 3.95715000
C 1.91169700 -0.50977900 -1.84168400
N 0.91183500 -0.79006900 1.28052800
C 2.83488500 -2.17415300 -0.10110100
C -4.37117300 0.49692200 1.70629200
H -5.24061200 -0.04154000 1.27625300
H -3.72075200 -0.25707400 2.18929700
H -4.75868900 1.15477100 2.50311300
H 2.81246700 2.33673700 -1.21320700
O -3.69039500 1.28095500 0.76098600
H -3.30527500 0.68180600 0.06372700
H -0.07958600 4.88118500 0.75410100
N -2.61602800 -0.37624200 -1.19397000
C -3.61403800 -0.53706200 -2.25836100
H -3.56300300 0.35710800 -2.89645600
H -3.36235700 -1.39354100 -2.91684300
C -2.33385100 -1.61171200 -0.44507400
H -1.77386300 -1.32415800 0.45525400
H -3.29351600 -2.01937600 -0.09144600
C -1.38745700 0.28172200 -1.66310500
H -0.60820100 0.10498400 -0.91006800
H -1.01724300 -0.18649000 -2.59594000
C -1.54097200 1.78398900 -1.86567200
H -0.57274600 2.21814300 -2.15754000
H -2.27011900 2.03035800 -2.65409100
H -1.86045600 2.27115800 -0.93356800
C -1.55717100 -2.69998400 -1.19112800
H -0.56349200 -2.34397000 -1.50195300
H -1.40564300 -3.56395300 -0.52537100
H -2.09078300 -3.05741000 -2.08589700
C -5.04008900 -0.67453800 -1.73476100
H -5.74982500 -0.74353600 -2.57381700
H -5.17123100 -1.57610900 -1.11653700
H -5.30384500 0.20054500 -1.12211500
```

5.1 Mn-CO_2-TBG (-1,1)

```
Mn -2.21096700 -1.13032300 -0.46564500
N -2.96739400 0.10221200 0.95031300
C -1.92712000 1.74663600 -0.40308300
C -2.64257100 1.42199600 0.81727300
C -3.68219400 -0.27021300 2.03671400
C -3.01970800 2.37126700 1.78696900
H -2.74337800 3.41727800 1.65745500
C -4.07918800 0.61238100 3.02571900
H -4.64807500 0.24356500 3.88054700
C -3.73457800 1.97148300 2.90335000
O -0.31821900 -2.59897100 -2.19724600
O -4.57716200 -1.53808100 -2.18745200
C -3.65787300 -1.33870100 -1.50096100
O -2.63749800 -3.55098800 1.17761900
C -1.02803200 0.89648700 -2.37864800
H -0.82813000 0.01615800 -2.98960300
C -0.66208000 2.15650700 -2.81828300
H -0.16115700 2.26656900 -3.78110700
C -1.58685200 3.05550700 -0.79710600
H -1.83011300 3.89690500 -0.14919600
C -0.94953600 3.26977200 -2.00715700
H -0.67690700 4.27857200 -2.32195100
C -2.47897000 -2.60075600 0.52566900
N -1.64485700 0.67216300 -1.19572800
C -1.07465700 -2.02557400 -1.52372600
H -3.93177300 -1.32908800 2.10802900
H -4.02779200 2.69559500 3.66575600
C -0.22478100 -0.97489200 0.92461100
O -0.07417900 0.17223400 1.32718800
O 0.39374100 -2.02878100 1.07150900
C 3.29731300 0.72805900 0.19725800
N 3.80890100 -0.07340100 1.06321700
N 3.09938600 0.49889100 -1.17022500
```

N 2.90285400 1.99754200 0.61702500
C 1.66074100 2.58040700 0.13466200
H 0.84476100 2.43315900 0.86112900
H 1.78102300 3.66015400 -0.05816800
H 1.34824800 2.09603000 -0.79343400
C 3.27834400 2.42087100 1.94810800
H 4.31817400 2.13941400 2.15614400
H 3.17097700 3.51483600 2.02408300
H 2.64529500 1.95443200 2.72877800
C 3.46787900 1.47966300 -2.16505000
H 4.13630800 1.03014400 -2.92443900
H 2.58917300 1.89112000 -2.69975500
H 3.99910800 2.31317400 -1.68842300
C 2.37035900 -0.65273600 -1.64934300
H 3.00369000 -1.35528900 -2.22663400
H 1.91549800 -1.19457300 -0.81304900
H 1.55417800 -0.32677700 -2.31766700
C 4.67632600 -1.22221200 0.80030600
C 5.62181100 -1.28619300 2.01909700
C 3.86802700 -2.53480000 0.76158400
C 5.54747600 -1.09427200 -0.46578400
H 6.21864400 -0.36280100 2.08817400
H 5.03406200 -1.37816100 2.94539700
H 6.31149700 -2.14327200 1.95149800
H 3.31298400 -2.64383900 -0.17826700
H 4.53717500 -3.40458500 0.86257800
H 3.13191900 -2.55080300 1.57816500
H 6.28433300 -1.91318700 -0.49703100
H 4.95804500 -1.13518800 -1.38942700
H 6.09989600 -0.14075700 -0.45671700

5.2 Mn-TBG-CO_2 (-1,1)

Mn -2.14003500 -1.13210500 -0.18224000
N -2.28634800 0.13623700 1.33529600
C -2.36639800 1.74571200 -0.38066200
C -2.42769900 1.48556300 1.01316200
C -2.31320000 -0.18050400 2.67005500
C -2.59096000 2.47051600 2.02195400
H -2.70075800 3.51526400 1.72744500
C -2.45632000 0.73944500 3.67865800
H -2.45640600 0.40053300 4.71638600
C -2.60304700 2.11901300 3.35171300
O -0.62518700 -2.45179500 -2.35200100
O -4.76553000 -2.24950200 -0.88492500
C -3.73557900 -1.78118300 -0.58937300
O -1.29400500 -3.36413900 1.56805300
C -2.07981500 0.77700700 -2.50513100
H -1.92430700 -0.13272900 -3.08654100
C -2.17050600 1.99570200 -3.13345600
H -2.08145100 2.04844700 -4.21998000
C -2.47890100 3.02588200 -0.98558300
H -2.64017300 3.89899800 -0.35190600
C -2.38176600 3.16647400 -2.35074600
H -2.46306400 4.14820500 -2.82141300
C -1.64352300 -2.48184800 0.88376100
N -2.16683000 0.60575600 -1.15107100
C -1.23180100 -1.92954700 -1.49722800
H -2.20481600 -1.23933800 2.90699400
H -2.72219200 2.87211000 4.13301000
C 4.28176800 2.45168100 1.19967200
O 3.98842400 3.47457800 0.72979500
O 4.59578500 1.43809000 1.67552000
C 2.28529800 0.01769300 -0.39549000
N 2.05963600 -0.89898300 0.47778900
N 2.99498000 -0.09927800 -1.59821100
N 1.78433100 1.29692800 -0.16023200
C 1.27195700 2.12406000 -1.24181400
H 0.17504100 2.21164800 -1.18101000
H 1.70032500 3.14022700 -1.19760900
H 1.52214500 1.68116000 -2.21163800
C 1.20627600 1.58794700 1.13855600
H 1.79699100 1.10402100 1.92479300
H 1.20277300 2.67834100 1.29433600
H 0.16849600 1.22238500 1.22150800
C 4.01668900 0.84334700 -1.99364000
H 5.01774900 0.36955000 -2.00754700
H 3.82538500 1.24860800 -3.00469300
H 4.04687000 1.69054800 -1.30005200
C 2.70644100 -1.15401200 -2.54232600
H 3.51005200 -1.91295100 -2.61238800
H 1.77143800 -1.65917000 -2.27451700
H 2.57826000 -0.72174800 -3.55225300
C 2.85105400 -2.10679900 0.71600700
C 2.80956500 -2.30630000 2.24696400
C 2.19763400 -3.34297900 0.06790700
C 4.33099900 -1.99618200 0.29546900
H 3.27716200 -1.44904000 2.75627300
H 1.76504800 -2.37638100 2.58642500
H 3.34113900 -3.22379600 2.54788100
H 2.21449500 -3.28963800 -1.02775500
H 2.73104800 -4.25867900 0.37060100
H 1.15015500 -3.43408300 0.38965200
H 4.89483300 -2.85687100 0.68971600
H 4.46330700 -1.98250800 -0.79294900
H 4.77603500 -1.07709200 0.70611600

5.3 Mn-MeOH-TBG (-1,1)

Mn -2.98491500 0.29924500 -0.45179200
N -2.09104600 -1.30081400 0.33981300
C -1.28443300 0.26847500 1.88697000
C -1.33866800 -1.09402000 1.48145100
C -2.18711900 -2.57519800 -0.13319300
C -0.70195000 -2.17893700 2.13758200
H -0.12987400 -1.99131200 3.04648700
C -1.57453100 -3.65741900 0.45625000
H -1.68878200 -4.64889300 0.01511000
C -0.80713500 -3.45705100 1.63470300
O -3.86336700 2.91134700 -1.53634300
O -5.72993400 -0.71857200 -0.14754800
C -4.64024300 -0.30218800 -0.23136100
O -2.54928400 -0.73520300 -3.18243100
C -2.00630900 2.45335000 1.41009900
H -2.58920400 3.11366000 0.76806400
C -1.31135500 2.96825200 2.47568800
H -1.35133000 4.04184700 2.66979400
C -0.55465900 0.75495100 2.99967700
H 0.00931000 0.04981500 3.61136200
C -0.55133000 2.09720500 3.30618200
H 0.01235000 2.48052000 4.15855700
C -2.72110800 -0.30870900 -2.10612500
N -2.02013800 1.12329000 1.06695900
C -3.51870100 1.88226000 -1.10370600
C 0.67871800 1.97063200 -0.74021100
H 0.39907900 2.92494300 -0.25326300
H 0.61462800 1.17995400 0.02619900
H 1.72822000 2.02517700 -1.06337700
H -2.78979300 -2.69852300 -1.03436800
O -0.12548100 1.68586700 -1.86749300
H -0.98913800 1.35298400 -1.54735500
H -0.31561900 -4.29524200 2.13229000
C 3.06620900 -0.49076600 -0.64684500
N 3.80383800 0.53372900 -0.90922400
N 3.00874700 -1.21026600 0.55394700
N 2.21656700 -0.97778300 -1.62860100
C 0.90509800 -1.52110900 -1.31378400
H 0.11608700 -0.85527900 -1.69658200
H 0.76527900 -2.52102300 -1.75975000
H 0.77486900 -1.60586400 -0.23169000
C 2.36375600 -0.51961500 -2.99388300
H 3.41668300 -0.29268800 -3.19799000
H 2.01447200 -1.30797000 -3.68205800
H 1.76547500 0.39044300 -3.18189300
C 3.06322200 -2.65520500 0.58082800
H 3.90087600 -3.00092500 1.21662700
H 2.13250800 -3.10317800 0.97689900
H 3.22405700 -3.03861000 -0.43466700
C 2.79408100 -0.55361600 1.82076300
H 3.68077900 -0.57762700 2.48436800
H 2.50972100 0.49308500 1.66067000
H 1.96729400 -1.04659000 2.36038800
C 5.06371300 0.89629400 -0.26084300
C 5.94495100 1.46646900 -1.39407600
C 4.84778400 2.01840400 0.77381600
C 5.83234900 -0.27845200 0.37905500
H 6.14070800 0.69037400 -2.15093000
H 5.42657100 2.30032200 -1.89172200
H 6.91191200 1.83017400 -1.00981100
H 4.27861000 1.66838300 1.64467500
H 5.81444800 2.40431600 1.13587000
H 4.29484500 2.85386200 0.31666700
H 6.84468500 0.05043900 0.66394800
H 5.34304900 -0.66975300 1.27853600

H 5.93479200 -1.10660100 -0.34021000

5.4 Mn-TBG-MeOH (-1,1)

Mn -2.58554800 -0.77816900 0.01805400
N -1.48077100 -0.21676800 1.56220000
C -1.53483500 1.87986100 0.48898800
C -1.09057700 1.12234000 1.60609200
C -1.08678900 -0.99949100 2.61964100
C -0.33454600 1.63625700 2.68992900
H -0.05515800 2.69060700 2.68602000
C -0.34872900 -0.54610800 3.68394800
H -0.07507100 -1.24200300 4.47935900
C 0.04515000 0.82203700 3.73228500
O -2.55197000 -1.48840900 -2.84889000
O -5.50789600 -0.73876800 0.34399500
C -4.34178100 -0.73935200 0.24699300
O -2.22542900 -3.64865900 0.64327500
C -2.76861400 1.79948500 -1.51082500
H -3.36903800 1.19571800 -2.19313000
C -2.52234800 3.12500600 -1.78107900
H -2.93136100 3.57565000 -2.68700900
C -1.25750100 3.25283700 0.25816000
H -0.65458700 3.80213200 0.98220400
C -1.73623900 3.88220100 -0.86863900
H -1.52155600 4.93629000 -1.05539300
C -2.38807000 -2.51438900 0.40798900
N -2.29053900 1.14610100 -0.41199700
C -2.57097800 -1.21254800 -1.71107000
C 5.02969600 -2.49886500 -0.72735400
H 4.17109600 -2.93160800 -1.28289900
H 5.41733400 -1.65744600 -1.33855200
H 5.81861400 -3.27051000 -0.69677200
H -1.39586700 -2.04374700 2.57555500
O 4.69934900 -2.11914000 0.58085700
H 4.01554100 -1.38801200 0.49842300
H 0.62865700 1.21099200 4.56845800
C 2.11990100 -0.32513300 -0.85453400
N 2.98484000 -0.10092800 0.08587000
N 1.83150500 0.50057200 -1.93554400
N 1.40004500 -1.50230600 -0.81661200
C 1.05703000 -2.23297600 -2.02384500
H 1.57316700 -3.21032400 -2.03999700
H -0.02825400 -2.41242900 -2.08837700
H 1.37145100 -1.67034900 -2.90980400
C 1.33008700 -2.26603300 0.41458900
H 1.26632300 -1.58302200 1.26918000
H 0.42767000 -2.89322900 0.39574900
H 2.21850900 -2.90716200 0.55725000
C 0.47040200 0.78224700 -2.34152600
H 0.29014300 1.87220000 -2.34886900
H 0.24479500 0.39464600 -3.35275700
H -0.23480800 0.33011400 -1.63538600
C 2.87764100 1.03787100 -2.77423800
H 2.96458900 2.13898400 -2.70810300
H 3.84319400 0.59707800 -2.49958400
H 2.67486300 0.78731700 -3.83201800
C 3.46382000 1.20003500 0.56919600
C 3.55564000 1.04073500 2.10270400
C 4.88360700 1.47729200 0.03874200
C 2.52133500 2.38062100 0.27310200
H 2.55963900 0.84377100 2.52569200
H 4.21347300 0.19571900 2.35734600
H 3.95988600 1.95195300 2.57244900
H 4.88594900 1.68406900 -1.03977300
H 5.32671300 2.34664200 0.55012700
H 5.52163600 0.59980100 0.22437300
H 2.84256800 3.26199300 0.85072200
H 2.50779000 2.66440800 -0.78564000
H 1.49300500 2.13349900 0.57302100

6.1 Mn-CO_2-TMEDA (-1,1)

Mn -2.45093300 -0.47451200 -0.10206600
N -0.95019500 -0.77009200 1.23234800
C -0.56819500 1.52972200 0.79500100
C -0.16401700 0.31598000 1.48375500
C -0.65267500 -1.93153200 1.85786100
C 0.91822400 0.24481000 2.37948000
H 1.52549700 1.13003500 2.55946600
C 0.40920400 -2.07117000 2.73523700
H 0.59894500 -3.03869200 3.20246700
C 1.21670100 -0.95236100 3.00831000
O -4.00806500 0.18269200 -2.52750900
O -4.72182000 -0.27811600 1.77983900
C -3.80278900 -0.33227800 1.06615600
O -2.75578900 -3.36578000 -0.63519900
C -2.18737800 2.51428300 -0.55957200
H -3.10611500 2.38539000 -1.13247900
C -1.56092500 3.74755600 -0.50102000
H -1.99528500 4.59840400 -1.02827600
C 0.12562900 2.75053100 0.88400100
H 1.07108300 2.79212200 1.42097100
C -0.36517800 3.86816900 0.23000800
H 0.16820000 4.81944700 0.27890000
C -2.64897600 -2.22754100 -0.41811200
N -1.70902200 1.41068000 0.05771200
C -3.40648100 -0.07635300 -1.56551800
H -1.30037000 -2.77905300 1.63195200
H 2.05928200 -1.02339300 3.69859500
C -0.69126100 -0.72212800 -1.74541400
O 0.32663500 -0.14161000 -1.36912500
O -0.97192800 -1.37194200 -2.75049500
N 3.22725100 1.80003300 0.02919400
C 3.26087000 0.34643100 0.12322000
H 3.44620700 0.08697300 1.17687000
H 2.26342400 -0.03226800 -0.14078200
C 4.28332700 -0.37184400 -0.77175900
H 4.21320400 0.03669200 -1.79323000
H 5.32235500 -0.16292500 -0.42109700
N 4.03766700 -1.80660500 -0.85834400
C 2.72561600 2.30447800 -1.23398000
H 1.82329500 1.74533700 -1.51754300
H 2.45699200 3.36926500 -1.13253900
H 3.46068400 2.23072200 -2.06823300
C 4.43073200 2.47024400 0.47185400
H 4.74072200 2.08067200 1.45509700
H 5.29530800 2.35920900 -0.22154500
H 4.24106000 3.55171100 0.57940800
C 4.88689300 -2.43434600 -1.85131300
H 4.62708400 -3.49984100 -1.95705600
H 4.74839700 -1.94955000 -2.83072000
H 5.97199000 -2.37645300 -1.59887400
C 4.12616800 -2.48352300 0.42290300
H 5.12741200 -2.37551300 0.90340300
H 3.37226200 -2.10047000 1.12548100
H 3.93658300 -3.55985900 0.28838600

6.2 Mn-TMEDA-CO_2 (-1,1)

Mn 1.78410200 -1.35267000 -0.30502800
N 2.32771500 0.57020000 -0.22535900
C 1.22979800 0.49989600 1.84503400
C 1.95356400 1.27818100 0.90159000
C 2.99427300 1.24047400 -1.20620100
C 2.28702100 2.65130800 1.03758000
H 1.98750400 3.18539400 1.93974300
C 3.33030000 2.57271100 -1.12721500
H 3.86640300 3.04532100 -1.95198300
C 2.97083800 3.30442100 0.03755500
O 0.82115100 -4.14237200 -0.06878200
O 4.41739700 -2.21094700 -1.31999500
C 3.38125000 -1.87903700 -0.88777300
O 0.49999900 -1.04057100 -2.93266500
C 0.34828500 -1.62720100 2.32593300
H 0.21241200 -2.66307700 2.01551500
C -0.18022200 -1.19410900 3.51491700
H -0.73213300 -1.89831700 4.14078700
C 0.70724300 0.98801500 3.06931800
H 0.87133300 2.03419000 3.33244100
C -0.00024400 0.16266600 3.91362300
H -0.40904000 0.53278100 4.85554600
C 0.99371600 -1.16714400 -1.87739300
N 1.05655500 -0.83162000 1.45505800
C 1.21089900 -3.04409000 -0.15512100
H 3.25933600 0.64695000 -2.08331100
H 3.22705300 4.36149000 0.13428000
C -5.49996900 -0.63079000 0.07398600
O -5.59970400 0.46293800 0.47173200
O -5.60230500 -1.74586700 -0.25912800
N -0.90983900 2.75300500 -0.77607600
C -1.24478800 1.33539000 -0.85408100
H -0.98293100 0.98428200 -1.86387000
H -0.58482900 0.78956500 -0.16321600

C -2.71456200 0.98204300 -0.55067000
H -2.96305100 1.29502600 0.47651700
H -3.38179300 1.55245700 -1.21797900
N -3.07243900 -0.42823400 -0.67343100
C -1.09321700 3.34804400 0.53091400
H -0.68705600 2.67906200 1.30254200
H -0.54640100 4.30402400 0.59097700
H -2.15557000 3.56064500 0.78856700
C -1.44198500 3.56722400 -1.84670700
H -1.22231500 3.10170500 -2.82089200
H -2.54253600 3.73422900 -1.79484100
H -0.96677300 4.56283900 -1.83265600
C -3.05058300 -0.94215200 -2.03347700
H -3.55954100 -1.91904900 -2.06045500
H -3.59855800 -0.25660400 -2.69898200
H -2.02995800 -1.07865800 -2.44146600
C -2.37608800 -1.30302900 0.25978900
H -1.30785600 -1.45217700 0.01104800
H -2.42763300 -0.88208500 1.27519800
H -2.86804600 -2.28908100 0.27194100

6.3 Mn-MeOH-TMEDA

Mn 0.92508300 -0.79149300 -1.01301100
N 0.63355000 1.16210300 -0.95612700
C 2.60753900 1.15362400 0.33012400
C 1.56874800 1.92743300 -0.25364700
C -0.38726500 1.85946100 -1.55940300
C 1.44537700 3.33513700 -0.14910600
H 2.19491600 3.88915000 0.41834800
C -0.54183000 3.22011900 -1.48579500
H -1.38958600 3.68680200 -1.99096000
C 0.39991300 3.99786900 -0.75169300
O 0.51681300 -3.19843500 0.64466700
O 2.53052600 -2.11047800 -3.09652300
C 1.89266900 -1.55653400 -2.28644800
O -1.64898300 -1.31787500 -2.37981200
C 3.43578000 -1.02956800 0.61290600
H 3.30450900 -2.09148300 0.39819800
C 4.49961700 -0.58925000 1.36576900
H 5.22262200 -1.30903100 1.75316400
C 3.68646800 1.65861300 1.10254900
H 3.75829100 2.73266500 1.27869400
C 4.63183400 0.80314200 1.62246400
H 5.46389500 1.18490400 2.21761700
C -0.63502300 -1.10648900 -1.84041800
N 2.48371300 -0.20431900 0.09294100
C 0.66274900 -2.23863600 -0.00866100
C 0.86374400 -0.30730600 3.01269800
H 0.86719900 -1.39569100 2.82270900
H 1.89997500 0.05791600 2.88907800
H 0.56995400 -0.14747000 4.06280300
H -1.10383100 1.25931300 -2.11891900
O -0.05407100 0.38096800 2.19330100
H 0.11161500 0.11704600 1.26644300
H 0.29729300 5.08175200 -0.67438500
N -2.68233600 -2.01409000 0.76722300
C -3.63181200 -0.95666300 0.47288200
H -3.92222500 -1.06251700 -0.58286900
H -4.56889600 -1.05084400 1.07290300
C -3.05117200 0.44090500 0.69669100
H -2.63345200 0.50649700 1.71034800
H -2.19165300 0.58295300 0.01037100
N -4.04456800 1.50063400 0.55234800
C -2.99615600 -3.26161900 0.10766400
H -3.08312400 -3.09983700 -0.97708100
H -2.18292600 -3.98430400 0.27729800
H -3.94559900 -3.72639600 0.46660400
C -2.41338300 -2.19050900 2.17856100
H -2.01887300 -1.26268900 2.61437600
H -3.31618800 -2.50137300 2.75703400
H -1.64066400 -2.96274700 2.30790000
C -3.58918800 2.74652900 1.14324800
H -4.38138800 3.50984600 1.07602000
H -3.35010500 2.59638100 2.20798400
H -2.68097100 3.15915800 0.64908300
C -4.46798000 1.69647700 -0.82290300
H -3.63668200 2.01973100 -1.49002400
H -4.88835000 0.76986100 -1.24040600
H -5.25341400 2.46756800 -0.86970900

6.4 Mn-TMEDA-MeOH (-1,1)

Mn -2.65106800 0.40746100 0.03277800
N -1.78666800 -1.38729700 -0.13327800
C -0.32701600 -0.51291400 1.48348000
C -0.65346100 -1.60287400 0.63025400
C -2.16439300 -2.37302200 -0.99753100
C 0.09119600 -2.80494800 0.50685900
H 1.00860200 -2.91561200 1.08284100
C -1.48326400 -3.55900700 -1.14746700
H -1.84602400 -4.30335900 -1.85857900
C -0.31150500 -3.78389600 -0.37361900
O -3.84706400 3.01707900 0.76347000
O -5.34094900 -0.71016700 -0.46808200
C -4.28015500 -0.26726500 -0.24547400
O -2.11778700 1.19000700 -2.74911900
C -0.93276600 1.64963400 2.17346100
H -1.62856200 2.48483900 2.09117800
C 0.14356300 1.73341900 3.02076500
H 0.28878000 2.64131800 3.60979800
C 0.79826700 -0.47139900 2.34275400
H 1.47431400 -1.32508100 2.35531700
C 1.05144100 0.63962300 3.11536200
H 1.92001600 0.68260100 3.77519500
C -2.30467100 0.89707400 -1.62998500
N -1.21375900 0.55925800 1.38513400
C -3.37619100 1.98500500 0.48150100
C 4.73234000 3.02444300 -0.29750800
H 4.00930200 3.86494100 -0.25090200
H 4.56638600 2.40314000 0.60688200
H 5.74258100 3.45981200 -0.21015800
H -3.06105200 -2.16640200 -1.58443100
O 4.65108600 2.29088700 -1.49084200
H 3.73460300 1.90374300 -1.54268000
H 0.26122600 -4.70734200 -0.48167100
N 3.31179300 -1.75548000 0.45203100
C 3.29131600 -0.41153300 -0.10589700
H 3.09556900 0.28104300 0.72633800
H 4.27929600 -0.12300300 -0.53014200
C 2.22579600 -0.19920100 -1.18291400
H 2.50710700 -0.74630700 -2.09451000
H 1.25897100 -0.61144600 -0.83913300
N 2.10361400 1.21991600 -1.54169300
C 4.23616400 -1.84140100 1.56567200
H 3.97486900 -1.09394500 2.33120400
H 4.18251800 -2.83936100 2.02803400
H 5.29489600 -1.66057600 1.26771700
C 3.55647800 -2.79199900 -0.53303400
H 2.75231000 -2.81786600 -1.28208900
H 4.52595200 -2.66285800 -1.06779500
H 3.57408900 -3.77519900 -0.03818900
C 1.56309600 1.40416700 -2.88535600
H 1.56159900 2.47615000 -3.13676500
H 2.19380600 0.88032300 -3.61922000
H 0.52386100 1.03212000 -2.98013500
C 1.32072800 1.97695300 -0.56320100
H 0.25838200 1.66652600 -0.55141700
H 1.72186200 1.83264600 0.44800500
H 1.37252600 3.05021900 -0.80164200

Optimized geometries of the intermediates reported in Table 3.

Mn-H⁻···CO₂ (-1,2)

Mn -1.35742300 0.00101700 0.06329400
N 0.18534700 1.31505600 -0.37304800
C 1.39350400 -0.70902800 -0.66510100
C 1.39485800 0.70510000 -0.66577100
C 0.12079300 2.64884200 -0.37306900
C 2.53072400 1.51146900 -0.94860600
H 3.47612700 1.03878600 -1.17319000
C 1.19245800 3.47929900 -0.64030500
H 1.06682300 4.55198900 -0.62113800
C 2.43649300 2.87618300 -0.93522300
O -3.17948100 -2.07161800 1.08294700
O -2.45791700 0.00059900 -2.70120300
C -1.99192300 0.00063400 -1.64435800
O -3.17587300 2.07803400 1.08047800
C 0.11577000 -2.65006500 -0.37045200

```
H -0.85272400 -3.07485800 -0.14117900
C 1.18584600 -3.48281200 -0.63691700
H 1.05817900 -4.55524300 -0.61669200
C 2.52781700 -1.51782200 -0.94720900
H 3.47409100 -1.04714000 -1.17230600
C 2.43100500 -2.88234300 -0.93248400
H 3.29926600 -3.49263600 -1.14654000
C -2.46788600 1.26790400 0.65309800
N 0.18286200 -1.31640100 -0.37172000
C -2.47012500 -1.26323600 0.65453300
H -0.84690200 3.07569200 -0.14424700
H -0.79413600 0.00133400 1.57900200
H 3.30592000 3.48462100 -1.14982600
C 1.72506500 0.00183600 2.58547200
O 1.74592800 -1.15751100 2.60786800
O 1.74547900 1.16120700 2.60684900
```

(Mn···H^-···CO_2)^{TS} (-1,2)

```
Mn -1.22721300 0.01051500 -0.13894700
N 0.34113500 1.32423000 -0.36865300
C 1.58384100 -0.69527000 -0.46192900
C 1.58499600 0.72030400 -0.44205000
C 0.27004600 2.65936000 -0.38861000
C 2.74924000 1.52949700 -0.51431500
H 3.72121900 1.06012900 -0.56449400
C 1.36845800 3.49160600 -0.46210900
H 1.23751000 4.56374800 -0.47090500
C 2.64822500 2.89417700 -0.52148400
O -3.15758800 -2.07604400 0.63362800
O -2.01238100 0.06146600 -2.99872600
C -1.68441300 0.04188700 -1.89338500
O -3.14880100 2.07532300 0.71056500
C 0.26547600 -2.63277600 -0.46806600
H -0.73012900 -3.05465600 -0.43396700
C 1.36303200 -3.46458100 -0.55942700
H 1.23053100 -4.53587500 -0.59788200
C 2.74722700 -1.50419600 -0.55196600
H 3.72017000 -1.03521400 -0.58394300
C 2.64406900 -2.86794400 -0.59689400
H 3.53432800 -3.48015500 -0.66321600
C -2.39924500 1.27318000 0.35101500
N 0.33844100 -1.29898200 -0.41113200
C -2.40440500 -1.26433200 0.30437500
H -0.72480100 3.08156500 -0.33940000
H -0.80174800 -0.01883500 1.45687400
H 3.53914800 3.50659300 -0.57581700
C 0.49562600 -0.08924800 2.70508500
O 0.59556000 -1.25217600 2.85984700
O 0.69871300 1.05681900 2.88262400
```

(Mn···H-CO_2^-) (-1,2)

```
Mn 1.32528400 -0.00059400 -0.03374800
N -0.19150300 -1.29178900 -0.46105800
C -1.38727400 0.72739700 -0.76901400
C -1.38768300 -0.72756400 -0.76857100
C -0.10262000 -2.63480700 -0.45956300
C -2.50514000 -1.50812300 -1.06603300
H -3.44662900 -1.03824000 -1.30525200
C -1.16992000 -3.45943900 -0.74241800
H -1.03740900 -4.53140900 -0.71921400
C -2.40246800 -2.88448400 -1.05014700
O 3.01285600 2.03550300 1.28820700
O 2.90826900 -0.00077100 -2.52276100
C 2.26856600 -0.00078600 -1.55889800
O 3.01246400 -2.03687100 1.28843500
C -0.10105300 2.63410100 -0.46152500
H 0.86664400 3.04825200 -0.22335500
C -1.16791400 3.45916400 -0.74479100
H -1.03476600 4.53107200 -0.72239600
C -2.50433300 1.50840600 -1.06679600
H -3.44612600 1.03890300 -1.30556000
C -2.40085900 2.88471800 -1.05186600
H -3.25980400 3.50163700 -1.27614200
C 2.34218500 -1.24708700 0.77256500
N -0.19074400 1.29114400 -0.46200800
C 2.34244700 1.24575600 0.77245100
H 0.86479300 -3.04936300 -0.22095100
H -0.37216700 -0.00161700 2.15699300
H -3.26172300 -3.50106200 -1.27417400
C -1.39908900 0.00141200 2.61086200
O -1.89317300 1.13105000 2.82952600
O -1.90019900 -1.12526100 2.82880100
```

(Mn H-CO_2^-)^{TS} (-1,2)

```
Mn -1.59549100 -0.02738900 0.19444800
N -0.12443800 1.30981400 -0.27111100
C 1.09669100 -0.67576800 -0.69151900
C 1.06219800 0.77966600 -0.66326500
C -0.24797500 2.64954300 -0.24976600
C 2.14030900 1.58977600 -1.01679700
H 3.08169200 1.14290600 -1.29283500
C 0.77796600 3.50389500 -0.59513200
H 0.61932000 4.57191500 -0.55569100
C 2.00252900 2.96351500 -0.98199000
O -3.20276200 -2.13463800 1.50587400
O -3.28860500 0.02891100 -2.22381900
C -2.60589700 0.00663000 -1.28976300
O -3.25793700 1.93333000 1.65594800
C -0.12569800 -2.61919000 -0.35943600
H -1.06780000 -3.06302900 -0.07625300
C 0.94229200 -3.40972700 -0.72831600
H 0.83491800 -4.48487200 -0.72948600
C 2.21437500 -1.41843400 -1.06786400
H 3.13667400 -0.91817300 -1.31361100
C 2.14243300 -2.79733100 -1.08328100
H 3.00332300 -3.38745000 -1.36536300
C -2.59489400 1.17257000 1.08793000
N -0.06588600 -1.27525400 -0.32895700
C -2.56015700 -1.31682600 0.99693800
H -1.20809800 3.03642100 0.05577000
H 3.32549000 1.36680500 1.78559300
H 2.83165000 3.60392400 -1.24892900
C 3.52877200 0.26855900 1.69077800
O 2.78536600 -0.48223800 2.36219600
O 4.46702800 -0.04622700 0.92342100
```

(Mn···$OCHO^-$) (-1,2)

```
Mn -1.02268000 -0.36737600 -0.26780000
N 0.23714100 1.25879900 -0.46842700
C 1.89597200 -0.38692000 -0.03554200
C 1.57797000 0.97652000 -0.26191900
C -0.12276500 2.52245500 -0.74321100
C 2.52561800 2.02899300 -0.30901800
H 3.56994200 1.81197000 -0.13674300
C 0.75953300 3.57627400 -0.80762100
H 0.40033800 4.57095200 -1.02713500
C 2.12961800 3.31188400 -0.57426700
O -2.60035300 -2.80794800 0.30887000
O -1.04759700 -0.84355400 -3.16653200
C -1.05204200 -0.66303700 -2.03036700
O -3.53542100 1.19429900 -0.36985200
C 1.06579400 -2.57700800 0.11737000
H 0.20040500 -3.22499400 0.08483900
C 2.31826800 -3.11018200 0.32403200
H 2.43865900 -4.17518700 0.45775300
C 3.20552700 -0.88486600 0.17736400
H 4.03927200 -0.19799500 0.19440800
C 3.42005600 -2.22446700 0.35853600
H 4.42201000 -2.59931300 0.52189900
C -2.55418900 0.59622000 -0.32741200
N 0.82723700 -1.26757400 -0.04869400
C -1.98126000 -1.86578000 0.08636600
H -1.17778500 2.68846900 -0.91392600
H -1.30848500 1.89514600 1.83825100
H 2.85771300 4.11169300 -0.60872700
C -0.98438900 1.05939200 2.49255300
O -0.81999500 -0.06139200 1.91844700
O -0.81513100 1.29462200 3.69694800
```

Optimized geometries of the intermediates reported in Table S4.

(Mn···CO_2) (a) (-1,1)

```
Mn 0.44266200 1.06709500 -0.36062700
```

```
N 0.64894900 -0.87349100 -0.69297800
C -1.66047700 -0.92475500 -0.26126100
C -0.49478700 -1.66062200 -0.58368400
C 1.80887100 -1.54035700 -0.99942700
C -0.44937200 -3.05648000 -0.76810700
H -1.36155300 -3.62822200 -0.66806200
C 1.89365500 -2.88809500 -1.18410700
H 2.85256700 -3.32958100 -1.41975900
C 0.72884900 -3.68723600 -1.06462700
O 0.49047500 2.52011100 2.19051900
O -0.33464100 3.35408700 -2.03828200
C -0.02712300 2.42874100 -1.39618300
O 3.30186500 1.66944100 -0.78011700
C -2.50106900 1.21308200 0.19766500
H -2.28212400 2.26656500 0.30143900
C -3.76968900 0.73586100 0.38176900
H -4.56849400 1.41918500 0.63387600
C -2.95289500 -1.46482600 -0.08231000
H -3.10320100 -2.52878300 -0.19808100
C -4.00645000 -0.65072700 0.23672200
H -4.99830100 -1.05928500 0.37572300
C 2.17494200 1.42195300 -0.62519500
N -1.43957900 0.42733000 -0.12055200
C 0.47530500 1.92242900 1.18740600
H 2.69199100 -0.92560500 -1.08360100
H 0.77048600 -4.75837300 -1.20562800
C 1.33450300 -1.00824200 2.37883000
O 0.21754900 -1.22879100 2.59922400
O 2.46736600 -0.82166100 2.21235000
```

$(Mn\cdots CO_2)$ (b) (-1,1)

```
Mn -0.48751600 -1.05307700 0.00000000
N -0.33297100 0.47423700 1.26497200
C -0.25440200 1.73968600 -0.70731900
C -0.25440200 1.73968600 0.70731900
C -0.35583300 0.40345000 2.62787200
C -0.17825700 2.89069200 1.52045800
H -0.11538500 3.86297600 1.05227000
C -0.28029000 1.48945000 3.45214200
H -0.29647800 1.34644500 4.52385400
C -0.18414600 2.78380300 2.88470700
O 0.41694800 -3.00615500 -2.01853900
O -3.29321500 -1.89509300 0.00000000
C -2.18729200 -1.52156100 0.00000000
O 0.41694800 -3.00615500 2.01853900
C -0.35583300 0.40345000 -2.62787200
H -0.43964000 -0.59073400 -3.04175100
C -0.28029000 1.48945000 -3.45214200
H -0.29647800 1.34644500 -4.52385400
C -0.17825700 2.89069200 -1.52045800
H -0.11538500 3.86297600 -1.05227000
C -0.18414600 2.78380300 -2.88470700
H -0.12393500 3.66244200 -3.51234400
C 0.05065600 -2.22979300 1.23005100
N -0.33297100 0.47423700 -1.26497200
C 0.05065600 -2.22979300 -1.23005100
H -0.43964000 -0.59073400 3.04175100
H -0.12393500 3.66244200 3.51234400
C 2.97080000 -0.43383100 0.00000000
O 2.76991500 0.70852200 0.00000000
O 3.25522700 -1.55984100 0.00000000
```

$(Mn^{-}\cdots CO_2)^{TS}$ (-1,1)

```
Mn 0.97491200 -0.41336500 -0.35965700
N -0.89169400 -1.12371400 -0.14803300
C -1.45372800 1.14848200 -0.30296000
C -1.90010100 -0.19831800 -0.14187400
C -1.22701100 -2.42839600 -0.01685000
C -3.24283900 -0.59306000 0.00688200
H -4.02225500 0.15487700 0.00792300
C -2.51739600 -2.86642100 0.13586300
H -2.71550300 -3.92376400 0.24072400
C -3.55953300 -1.92045400 0.15026000
O 3.59449800 0.94027800 -0.34431300
O 1.49332200 -1.67696400 -2.95743300
C 1.25879500 -1.14815500 -1.94618400
O 2.16190800 -2.53807000 1.30475500
C 0.40085900 2.53390300 -0.61308700
H 1.47093700 2.61188000 -0.73053000
C -0.38112600 3.65517500 -0.63809200
H 0.08323700 4.62158400 -0.77850800
C -2.29701500 2.27154000 -0.31945800
H -3.36266300 2.13467100 -0.20445400
C -1.77691600 3.53153000 -0.47950100
H -2.41846300 4.40158200 -0.49246700
C 1.68588600 -1.69407700 0.65667000
N -0.08559000 1.27204400 -0.43166900
C 2.55771300 0.41809400 -0.35905500
H -0.40362200 -3.12749900 -0.03906900
H -4.58839400 -2.23255800 0.26753900
C 0.71199700 0.58748200 2.26769300
O -0.38818100 0.25940700 2.55801800
O 1.74688100 1.12423400 2.48601600
```

$(Mn\text{-}COO^{-})$ (-1,1)

```
Mn -1.04209000 -0.00495700 0.32507600
N 0.55127900 -1.29509300 0.26746700
C 1.76142600 0.73352100 0.09654600
C 1.76748400 -0.72031500 0.09476900
C 0.48316400 -2.63688400 0.32618300
C 2.92397500 -1.49446900 -0.03941700
H 3.88247900 -1.01749000 -0.17583200
C 1.58464400 -3.45473700 0.19664600
H 1.46368600 -4.52738800 0.24623900
C 2.83711600 -2.87003400 0.00314900
O -3.05294500 2.06720500 -0.26513700
O -1.56538800 -0.02284600 3.22941700
C -1.30663400 -0.01489500 2.09934700
O -3.03152100 -2.08898100 -0.29515900
C 0.46182400 2.63905200 0.33355100
H -0.52410400 3.05135600 0.48789400
C 1.55645900 3.46605600 0.20454700
H 1.42679800 4.53757600 0.25662500
C 2.91133900 1.51736500 -0.03741100
H 3.87337600 1.04833700 -0.17626300
C 2.81346000 2.89208100 0.00815100
H 3.69481800 3.50933500 -0.09866700
C -2.24976500 -1.27488500 -0.03767000
N 0.54056400 1.29787400 0.27167000
C -2.26248700 1.25769300 -0.02036900
H -0.49962500 -3.05735900 0.47859800
H 3.72361300 -3.47988000 -0.10374700
C -0.82761500 0.00531800 -2.06165500
O 0.35415300 0.00342800 -2.41968000
O -1.87405000 0.01253900 -2.72617900
```

Supporting References

[1] a) M. Sandroni, G. Volpi, J. Fiedler, R. Buscaino, G. Viscardi, L. Milone, R. Gobetto, C. Nervi, *Catal. Today* **2010**, *158*, 22-28; b) L. Rotundo, J. Filippi, R. Gobetto, H. A. Miller, R. Rocca, C. Nervi, F. Vizza, *Chem. Commun.* **2019**, *55*, 775-777.

[2] J. Du, Z.-L. Lang, Y.-Y. Ma, H.-Q. Tan, B.-L. Liu, Y.-H. Wang, Z.-H. Kang, Y.-G. Li, *Chem. Sci.* **2020**, *11*, 3007-3015.

[3] L. Rotundo, C. Garino, E. Priola, D. Sassone, H. Rao, B. Ma, M. Robert, J. Fiedler, R. Gobetto, C. Nervi, *Organometallics* **2019**, *38*, 1351-1360.
[4] M. J. Frisch, G. W. Trucks, H. B. Schlegel, G. E. Scuseria, M. A. Robb, J. R. Cheeseman, G. Scalmani, V. Barone, B. Mennucci, G. A. Petersson, H. Nakatsuji, M. Caricato, X. Li, H. P. Hratchian, A. F. Izmaylov, J. Bloino, G. Zheng, J. L. Sonnenberg, M. Hada, M. Ehara, K. Toyota, R. Fukuda, J. Hasegawa, M. Ishida, T. Nakajima, Y. Honda, O. Kitao, H. Nakai, T. Vreven, J. A. J. Montgomery, J. R. Peralta, F. Ogliaro, M. Bearpark, J. J. Heyd, E. Brothers, K. N. Kudin, V. N. Staroverov, R. Kobayashi, J. Normand, K. Raghavachari, A. Rendell, J. C. Burant, S. S. Iyengar, J. Tomasi, M. Cossi, N. Rega, J. Millam, M. Klene, J. E. Knox, J. B. Cross, V. Bakken, C. Adamo, J. Jaramillo, R. Gomperts, R. E. Stratmann, O. Yazyev, A. J. Austin, R. Cammi, C. Pomelli, J. Ochterski, R. L. Martin, K. Morokuma, V. G. Zakrzewski, G. A. Voth, P. Salvador, J. J. Dannenberg, S. Dapprich, A. D. Daniels, O. Farkas, J. B. Foresman, J. V. Ortiz, J. Cioslowski, D. J. Fox, Revision D.01 ed., Gaussian, Inc., Wallingford CT, **2009**.
[5] a) S. Miertuš, E. Scrocco, J. Tomasi, *Chem. Phys.* **1981**, *55*, 117-129; b) M. Cossi, G. Scalmani, N. Rega, V. Barone, *J. Chem. Phys.* **2002**, *117*, 43-54.
[6] a) A. D. Becke, *J. Chem. Phys.* **1993**, *98*, 5648-5652; b) C. Lee, W. Yang, R. G. Parr, *Phys. Rev. B: Condens. Matter* **1988**, *37*, 785-789.
[7] a) F. Weigend, R. Ahlrichs, *Phys. Chem. Chem. Phys.* **2005**, *7*, 3297-3305; b) F. Weigend, *Phys. Chem. Chem. Phys.* **2006**, *8*, 1057-1065.
[8] S. Grimme, S. Ehrlich, L. Goerigk, *J. Comput. Chem.* **2011**, *32*, 1456-1465.
[9] J. Septavaux, C. Tosi, P. Jame, C. Nervi, R. Gobetto, J. Leclaire, *Nat. Chem.* **2020**, *12*, 202-212.
